# Ciliopathy-related B9 protein complex regulates ciliary axonemal microtubule posttranslational modifications and initiation of ciliogenesis

**DOI:** 10.1172/JCI196365

**Published:** 2025-10-30

**Authors:** Ruida He, Yan Li, Minjun Jin, Huike Jiao, Yue Shen, Qize Han, Xilang Pan, Suning Wang, Zaisheng Lin, Jingshi Li, Chao Lu, Dan Meng, Zongfu Cao, Qing Shang, Nan Lv, Kai Wan, Huafang Gao, Xu Ma, Haiyan Yin, Haishuang Chang, Liang Wang, Minna Luo, Junmin Pan, Chengtian Zhao, Muqing Cao

**Affiliations:** 1 International Peace Maternity and Child Health Hospital, Key Laboratory of Cell Differentiation and Apoptosis of Chinese Ministry of Education, Department of Pathophysiology, Shanghai Jiao Tong University School of Medicine, Shanghai, China.; 2 Institute of Evolution and Marine Biodiversity, Ocean University of China, Qingdao, China.; 3 Laboratory for Marine Biology and Biotechnology, Qingdao Marine Science and Technology Center, Qingdao, China.; 4 National Human Genetic Resources Center, National Research Institute for Family Planning, Beijing, China.; 5 Tianjin Key Laboratory of Food and Biotechnology, School of Biotechnology and Food Science, Tianjin University of Commerce, Tianjin, China.; 6 Rehabilitation Center, Children’s Hospital Affiliated to Zhengzhou University, Henan Children’s Hospital, Zhengzhou Children’s Hospital, Zhengzhou, China.; 7 School of Acupuncture and Tuina, Chengdu University of Traditional Chinese Medicine, Chengdu, China.; 8 Shanghai Institute of Precision Medicine, Shanghai Ninth People’s Hospital, Shanghai Jiaotong University School of Medicine, Shanghai, China.; 9 School of Life Sciences, Jiangsu Normal University, Xuzhou, China.; 10 MOE Key Laboratory of Protein Sciences, Tsinghua-Peking Center for Life Sciences, School of Life Sciences, Tsinghua University, Beijing, China.

**Keywords:** Cell biology, Genetics, Cytoskeleton, Genetic diseases

## Abstract

Ciliary dysfunction results in multiorgan developmental diseases, collectively known as ciliopathies. The B9D1-B9D2-MKS1protein complex maintains the gatekeeper function at the ciliary transition zone (TZ). However, the function of B9 proteins and the mechanisms underlying why different variants in the same B9 gene cause different ciliopathies are not fully understood. Here, we investigated the function of B9 proteins and revealed 2 critical functions. First, the B9 complex interacted with and anchored TMEM67 to the TZ membrane. Disruption of the B9-TMEM67 complex reduced posttranslational modifications of axonemal microtubules due to deregulation of tubulin-modifying enzymes within cilia. Second, B9 proteins localized to centrioles prior to ciliogenesis, where they facilitated the initiation of ciliogenesis. In addition, we identified *B9D2* variants in a cohort of patients with Joubert syndrome. We found that Joubert syndrome–associated *B9D2* variants primarily affected axonemal microtubule modifications without disrupting ciliogenesis, whereas the Meckel syndrome–associated *B9D2* variant disrupted both ciliogenesis and axonemal microtubule modifications. Thus, besides its role as a gatekeeper for ciliary membrane proteins, the B9 complex also controls axonemal microtubule posttranslational modifications and early stages of ciliogenesis, providing insights into the distinct pathologies arising from different variants of the same gene.

## Abstract 2

This work identifies novel functions of the B9 protein complex and provides new insights into B9-related genetic diseases.

## Graphical abstract

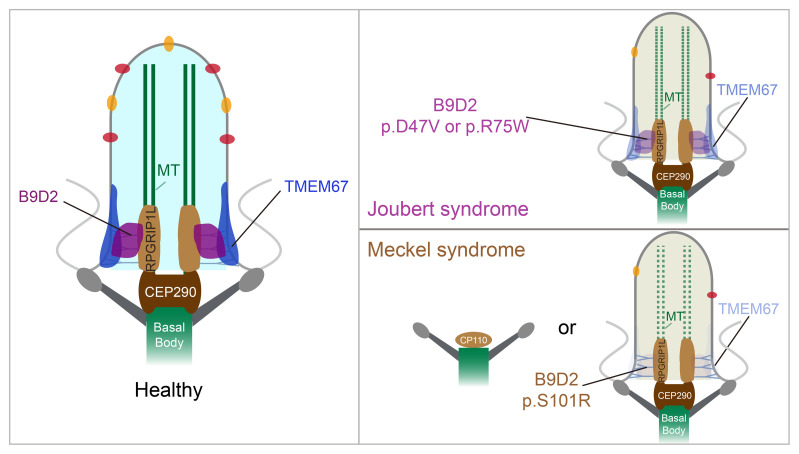

## Introduction

Primary cilia are highly conserved, antenna-like cellular organelles (1–4). In most nondividing mammalian cells, the mother centrioles recruit membrane vesicles, dock on the plasma membrane, and finally mature to the basal body, on which the axonemal microtubule with multiple posttranslational modifications is assembled to form primary cilium (5–9). Primary cilia are enriched with numerous signaling proteins and function as specialized platforms that coordinate various developmental signaling pathways (10–13). Ciliary defects underlie a wide spectrum of human diseases, collectively known as ciliopathies, which range from embryonic lethality to isolated organ abnormalities in adults. The clinical manifestations of ciliopathies include CNS malformations, renal cysts, retinal degeneration, polydactyly, infertility, and obesity (4, 11–13). Joubert syndrome (JBTS) and Meckel syndrome (MKS) are two ciliopathies that exhibit overlapping phenotypic and genetic features (14–16). Shared clinical manifestations of these two syndromes include brain malformations, cystic diseases, and polydactyly, though the pathogenic mechanisms controlling disease-associated developmental changes are not fully understood. A hallmark of JBTS is the malformation of the cerebellar vermis and mid-hindbrain, typically visualized on brain MRI as the “molar tooth sign.” This radiological feature is characterized by a deepened interpeduncular fossa; hypoplasia of the cerebellar vermis; and elongated, thickened, and maloriented superior cerebellar peduncles (15, 17). Common features of JBTS include developmental delay, respiratory dysregulation, abnormal ocular movements, hypotonia, and intellectual disability, often accompanied by oral-facial abnormalities, renal/hepatic cysts, retinal degeneration, and/or polydactyly (15, 16). In contrast, MKS is characterized by more severe developmental defects, including renal cysts, hepatic ductal plate malformation, polydactyly, and occipital encephalocele, and is generally lethal during the perinatal period, whereas most patients with JBTS exhibit a longer life expectancy, with a substantial proportion surviving into adulthood (15, 16, 18, 19).

Almost all MKS-associated genes are implicated in JBTS, reflecting the phenotypic overlap between the 2 conditions. Interestingly, most proteins encoded by these shared genes are localized to the ciliary transition zone (TZ), a specialized region at the base of the cilium that functions as a selective barrier to concentrate the ciliary membrane proteins in cilia (12, 15, 20–22). Reduced concentrations of ciliary membrane proteins have been observed in both MKS and JBTS, but it is not clear whether the changes in ciliary membrane proteins cause clinical features of the disease (16, 22–25). Several proteins associated with JBTS and MKS are located in the TZ, forming the MKS complex, which is positioned between the ciliary axoneme and the ciliary membrane (21, 22, 26–28). The MKS complex includes several membrane and membrane-associated proteins (e.g., TCTN1-3 and TMEM proteins), as well as 3 non-transmembrane proteins containing B9 domains (21–23, 26–28). The MKS complex is essential for the enrichment of signaling-related ciliary membrane proteins within cilia (20–24, 27, 29), although the functions of the MKS complex and the mechanisms utilized to assemble the MKS complex at the TZ are not fully understood. The B9 proteins — MKS1, B9D1 (MKS9), and B9D2 (MKS10) — form a subcomplex within the MKS complex at the TZ (20, 26, 29). These proteins are involved in the ciliary enrichment of membrane proteins, including ACIII, ARL13B, and INPP5E, and the loss of B9 components disrupts Hedgehog signaling, which is mediated by the ciliary membrane (20, 24, 29). Moreover, disruption of the B9 proteins substantially impairs ciliogenesis in animals through mechanisms not fully understood (20, 28–30). Consistent with the importance of the B9 complex, variants in *MKS1*, *B9D1*, or *B9D2* cause either MKS or JBTS in humans (16, 20, 24). The functions of B9 proteins and the reasons why different variants in the same B9 gene lead to either MKS or JBTS require further investigation.

In this study, we demonstrate that B9 proteins play dual roles in the early stages of ciliogenesis and in the regulation of ciliary axonemal microtubule posttranslational modifications. During ciliogenesis, B9 proteins localize to the mother centriole, facilitate the docking of membrane vesicles to the distal appendages, and promote the removal of CP110 from the mother centriole, thus initiating cilia assembly. We also found that the B9 complex interacts with and anchors TMEM67 to the TZ membrane, thereby stabilizing the MKS module and maintaining the integrity of the TZ diffusion barrier. Disruption of this barrier results in the leakage of cytosolic proteins into the cilia, impairs the posttranslational modifications of axonemal microtubules, and ultimately compromises ciliary stability. These findings suggest that defects in the TZ, leading to the aberrant distribution of nonmembrane proteins within cilia, may contribute to ciliopathies in a manner similar to the mislocalization of ciliary membrane proteins. Additionally, we identified *B9D2* variants in a screen of a Chinese JBTS cohort and showed that JBTS-associated variants in *B9D2* primarily impaired TZ integrity, while the MKS-associated variant in *B9D2* disrupted both ciliogenesis and TZ integrity. These findings not only reveal functions of B9 proteins but also provide insights into the pathogenesis of different ciliopathies.

## Results

### Loss of B9 proteins attenuates posttranslational modifications of axonemal microtubules.

To investigate the function of B9D2, we employed CRISPR/Cas9 technology to knock out *B9D2* in hTERT-RPE1 (RPE1) cells (Supplemental Figure 1, A and B; supplemental material available online with this article; https://doi.org/10.1172/JCI196365DS1). Consistent with previous reports in mammalian cells, ciliogenesis was compromised in *B9D2*-KO cells (Supplemental Figure 1, C–E) and 2 ciliary membrane proteins, ARL13B and INPP5E (31, 32), failed to accumulate in the cilia of *B9D2*-KO cells (Supplemental Figure 1, C and F) (20, 29). These results demonstrated successful KO of *B9D2*.

**Figure 1:**
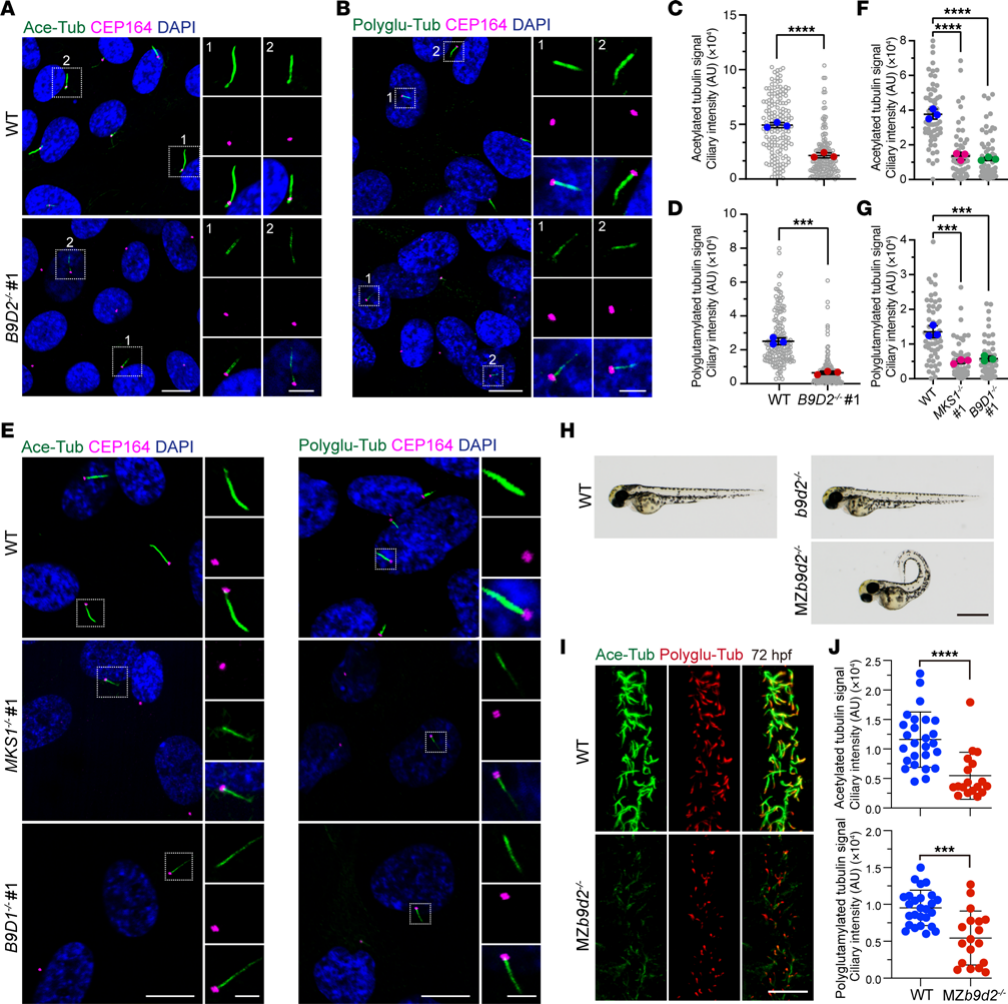
Loss of B9D2 attenuates posttranslational modifications of the axonemal microtubules. (**A** and **B**) Representative images of WT and *B9D2^–/–^* RPE1 cells stained for CEP164 (magenta), DAPI (blue), and acetylated tubulin (green) (**A**) or polyglutamylated tubulin (green) (**B**). Scale bars in low or high magnification view are 10 μm or 3 μm, respectively. (**C** and **D**) Quantification of fluorescent intensity of acetylated tubulin and polyglutamylated tubulin in cilia in **A** and **B** (experiments were done in triplicates). (**E**) Representative images of WT, *MKS1^–/–^*, and *B9D1^–/–^* RPE1 cells stained for CEP164 (magenta), DAPI (blue), and acetylated tubulin (green, left panel) or polyglutamylated tubulin (green, right panel). (**F** and **G**) Quantification of fluorescent intensity of acetylated tubulin and polyglutamylated tubulin in **E** in cilia (experiments were done in triplicates). (**H**) Representative images of WT and maternal-zygotic (MZ) *b9d2* mutant embryos (72 hours postfertilization [hpf]). Scale bar: 1 mm. (**I**) Representative images of the central canals in WT and MZ*b9d2* mutant embryos at 72 hpf stained for acetylated tubulin (green) and polyglutamylated tubulin (red). Scale bar: 10 μm. (**J**) Quantification of fluorescent intensity of acetylated tubulin and polyglutamylated tubulin in cilia in **I** (*n* = 6 WT fish and 6 MZ*b9d2^–/–^* fish). Data are presented as mean ± SD. ***P < 0.001, ****P < 0.0001 by 2-tailed unpaired Student’s *t* test (**C**, **D**, and **J**), by 1-way ANOVA with Dunnett’s test (**F** and **G**).

Unexpectedly, we observed a marked reduction in the acetylation of the axonemal microtubules in the cilia of *B9D2*-KO cells compared with WT cells (Supplemental Figure 1C). This prompted us to quantify the levels of acetylation and polyglutamylation, 2 key posttranslational modifications of axonemal microtubules. Similar to the decrease in acetylation, polyglutamylation was also reduced (Figure 1, A–D). Immunoblot analysis of global acetylation and polyglutamylation levels in whole cells revealed no changes in either modification (Supplemental Figure 1G), suggesting that these compromised modifications are specific to axonemal microtubules.

Given the importance of B9 complex integrity for its function, we hypothesized that MKS1 and B9D1 might play roles similar to B9D2. Using CRISPR/Cas9 technology, we generated *MKS1*-KO and *B9D1*-KO RPE1 cell lines (Supplemental Figure 1, H and I). As expected, loss of either MKS1 or B9D1 led to defects in ciliogenesis and a failure in the accumulation of ciliary membrane proteins (Supplemental Figure 1, J–L). Interestingly, both acetylation and polyglutamylation of axonemal microtubules were reduced in *MKS1*-KO and *B9D1*-KO cells (Figure 1, E–G), consistent with findings from the *B9D2*-KO cells. Global tubulin modifications remained unaffected in *MKS1*-KO and *B9D1*-KO cells (Supplemental Figure 1M). To confirm these results, we performed experiments in other B9 gene KO RPE1 cell lines generated with different sgRNAs and found consistent results (Supplemental Figure 2, A and B). Moreover, we observed the same phenotypes in mouse NIH-3T3 cells, suggesting that the regulatory mechanism of the B9 complex in posttranslational modifications of axonemal microtubules and in the accumulation of ciliary proteins is conserved across species (Supplemental Figure 2, C–E). Collectively, these results indicate that the B9 complex plays a pivotal role in regulating the posttranslational modifications of axonemal microtubules.

### b9d2 mutations reduce posttranslational modifications of the axonemal microtubules and cause ciliopathy phenotypes in zebrafish.

The zebrafish serves as an excellent model organism for studying ciliopathies (33, 34). Previous research demonstrated that suppression of *b9d2* using morpholino (MO) or KO of *b9d2* induces ciliopathy-related developmental defects in zebrafish (20, 35). We generated a *b9d2* mutant zebrafish line using the CRISPR/Cas9 method (Supplemental Figure 3, A and B). Consistent with previous studies (35), adult zebrafish mutants developed pronounced spinal curvature during later stages of development (Supplemental Figure 3C), indicating the successful generation of the zebrafish line. However, the ciliary number and acetylation levels of cilia in the mutant larvae were normal (Supplemental Figure 3, D and E). Given the potential effects of maternal proteins, we further generated maternal-zygotic *b9d2* mutants. Interestingly, maternal-zygotic mutant larvae (*MZb9d2*) displayed a severe dorsal curvature phenotype, in stark contrast to WT and zygotic mutants (Figure 1H). The number of cilia in the spinal canal of maternal-zygotic mutants was reduced compared with WT, although cilia length was comparable (Supplemental Figure 3, F and G). Notably, we observed a reduction in both acetylation and polyglutamylation of axonemal microtubules in the spinal canal of maternal-zygotic mutants (Supplemental Figure 3F and Figure 1, I and J). These findings indicate that *b9d2* plays conserved roles in regulating the posttranslational modifications of ciliary axonemal microtubules and ciliogenesis.

### The B9 complex anchors TMEM67 to the TZ, maintaining ciliary protein composition and tubulin modifications in cilia.

**Figure 2:**
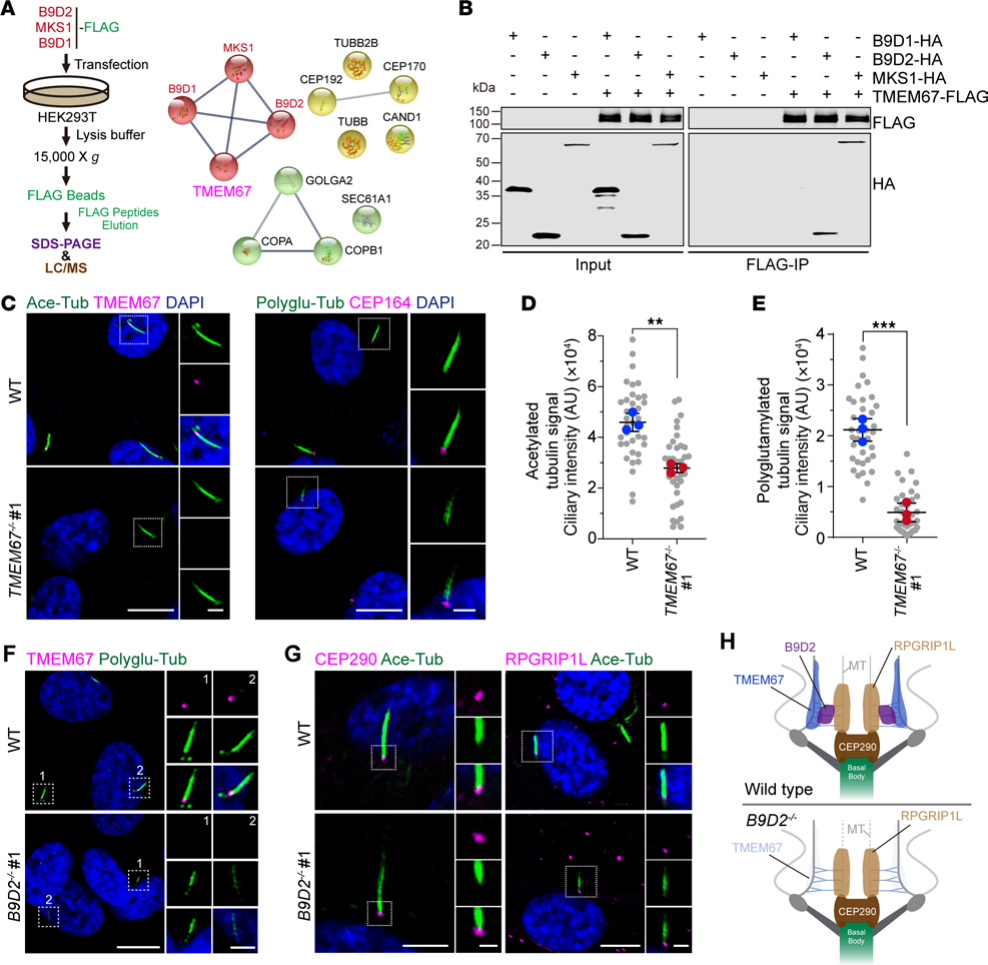
The B9 complex anchors TMEM67 at the TZ to maintain the modifications of axonemal microtubules. (**A**) Procedure for the purification and analysis of FLAG-MKS1, B9D1, and B9D2 from HEK293T cells. The proteins identified by liquid chromatography–mass spectrometry are clustered by STRING online tool. (**B**) Co-IP assay using protein lysates from HEK293T cells transfected with B9D1-HA, B9D2-HA, or MKS1-HA and TMEM67-FLAG plasmids to test the interaction between B9 proteins and TMEM67. Western blotting indicates the interactions between TMEM67 and B9D2 or MKS1. (**C**) Representative images of WT and *TMEM67^–/–^* cells stained for acetylated tubulin (green), TMEM67 (magenta), and DAPI (blue) in the left panel and polyglutamylated tubulin (green), CEP164 (magenta), and DAPI (blue) in the right panel. (**D** and **E**) Quantification of fluorescent intensity of acetylated tubulin and polyglutamylated tubulin in cilia in **C** (experiments were done in triplicates). (**F** and **G**) Representative images of WT and *B9D2^–/–^* cells stained for polyglutamylated tubulin (green), TMEM67 (magenta), and DAPI (blue) in **F** and acetylated tubulin (green), CEP290 (magenta, left panel), RPGRIP1L (magenta, right panel), and DAPI (blue) in **G**. (**H**) Schematic representation of the transition zone in WT and *B9D2^–/–^* cells. In this figure, the cells were treated with serum starvation for 48 hours before fixation. Scale bars in low or high magnification view are 10 μm or 2 μm, respectively, in **C**, **F**, and **G**. Data are presented as mean ± SD. **P < 0.01, ***P < 0.001 by 2-tailed unpaired Student’s *t* test (**D** and **E**).

The B9 proteins are soluble components localized between the ciliary membrane and microtubule axoneme at the TZ. To investigate the interactions within the MKS complex and the mechanism anchoring the B9 complex to the ciliary membrane, we performed co-IP using the B9 complex as bait to identify interacting proteins (Figure 2A). The pulldown products were analyzed via mass spectrometry, leading to identification of centrosomal and ciliary proteins as well as membrane trafficking–related proteins, clustered using the STRING online tool (Figure 2A). TMEM67, a ciliary membrane protein primarily localized at the ciliary TZ, is genetically linked to both MKS and JBTS (36). Functional and physical interactions among B9 proteins, TCTN proteins, TMEM231, and TMEM67 have been observed in mammalian cells and *C*. *elegans* previously (21, 23, 27, 28). Co-IP experiments using FLAG-TMEM67 as bait confirmed interactions between TMEM67, B9D2, and MKS1 (Figure 2B). Given the interactions between TMEM67 and the B9 complex, we hypothesized that B9 proteins form a subcomplex with TMEM67 to stabilize the MKS module at the TZ, and loss of any member of this B9-TMEM67 subcomplex might lead to similar phenotypes. To test this, we knocked out *TMEM67* using CRISPR/Cas9 (Supplemental Figure 4A and Figure 2C). Consistent with observations in B9 mutants, cells lacking TMEM67 exhibited attenuated ciliogenesis (Supplemental Figure 4B), along with reduced ARL13B and INPP5E in cilia (Supplemental Figure 4, C and D). Notably, the acetylation and polyglutamylation of axonemal microtubules were also diminished in *TMEM67*-KO cells (Figure 2, C–E). We observed consistent results from another *TMEM67*-KO RPE1 cell line generated with different sgRNA (Supplemental Figure 4, E and F).

Previous studies in mammalian cells have shown that several MKS components fail to localize to the TZ in the absence of either *MKS1*, *B9D2*, *Tmem231*, or *B9d1* (20, 23, 29). Given the biochemical interaction of TMEM67 with B9D2 and MKS1, we assessed whether B9D2 and MKS1 are required for TMEM67 localization at the TZ. In *B9D2*-KO and *MKS1*-KO cells, TMEM67 localization to the TZ was largely disrupted, unlike in WT cells (Figure 2F and Supplemental Figure 5, A and B). In contrast, the localizations of 2 nephronophthisis (NPHP) module components, CEP290 and RGPRIP1L, were unaffected (Figure 2G and Supplemental Figure 5, C and D). These results indicate that the B9 complex is essential for TMEM67 localization at the TZ (Figure 2H). Previous research showed that TMEM67 fails to concentrate to the ciliary base in the absence of TCTN1 or TMEM231, two other membrane-associated proteins binding TMEM67 at the TZ. Thus, proper TMEM67 localization also requires nonmembrane subunits situated between the microtubule axoneme and the ciliary membrane. It appears that TMEM67 is not essential for the proper localization of B9D2 and MKS1 because these 2 proteins exhibited only reduced concentrations at the TZ in *TMEM67*-KO cells compared with WT cells, while loss of any one of the B9 members led to mislocalization of the other 2 B9 proteins at the TZ (Supplemental Figure 5, E–G). Taken together, these data demonstrate that the B9 complex interacts with the ciliary membrane protein TMEM67 and stabilizes TMEM67 to the TZ, and they form a functional module to maintain posttranslational modifications of axonemal microtubules.

### The B9-TMEM67 subcomplex excludes cytosolic enzyme out of cilia to maintain cilium stability.

**Figure 3:**
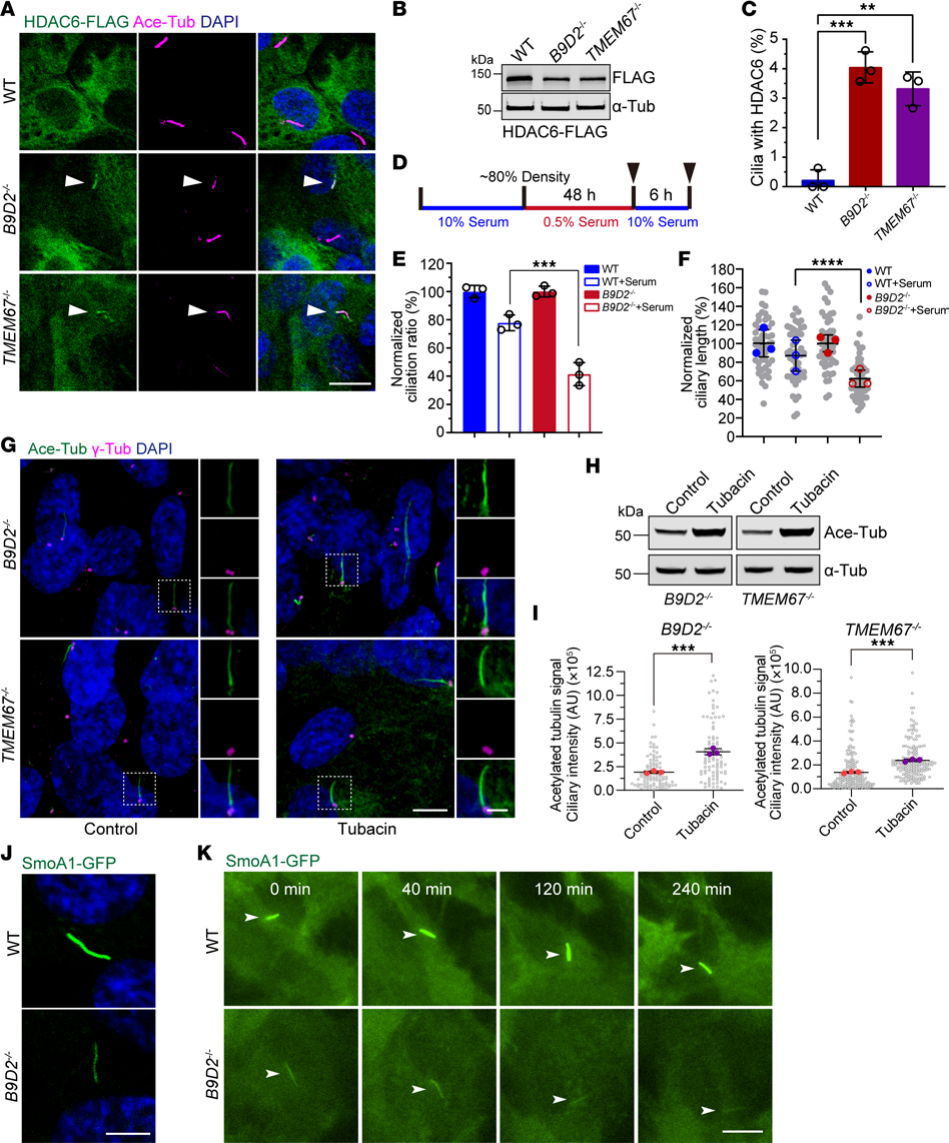
Disruption of the B9-TMEM67 complex fails to exclude HDAC6 out of cilia and attenuates ciliary axonemal stability. (**A**) Representative images of WT, *B9D2^–/–^*, and *TMEM67^–/–^* cells expressing FLAG-tagged HDAC6 stained for FLAG (green), acetylated tubulin (magenta), and DAPI (blue). Scale bar: 10 μm. (**B**) Western blot analysis of cells in **A** probed with antibodies against FLAG and α-tubulin. (**C**) Quantification of HDAC6-positive cilia in the cells in **A**. *n* = 3 replicates. (**D**) Schematic of the serum stimulation assay to induce ciliary assembly. Arrowheads indicate the time for fixing the cells. (**E**) Quantification of WT and *B9D2^–/–^* cells with cilia in the serum stimulation assay. *n* = 3 replicates. (**F**) Quantification of ciliary length of WT and *B9D2^–/–^* cells in the serum stimulation assay (experiments were done in triplicates). The ciliary lengths of serum-stimulated cells are normalized to nontreated controls. (**G**) Representative images of *B9D2^–/–^* and *TMEM67^–/–^* cells, treated with or without tubacin (2 μM) for 24 hours, stained for acetylated tubulin (green), γ-tubulin (magenta), and DAPI (blue). Scale bars in low or high magnification view are 5 μm or 2 μm, respectively. (**H**) Western blot analysis of the cells in **G**, probed with antibodies against acetylated tubulin and α-tubulin. (**I**) Quantification of fluorescent intensity of acetylated tubulin in cilia in **G** (experiments were done in triplicates). (**J**) Representative images of localization of SmoA1 in WT and *B9D2^–/–^* cells. Scale bar: 4 μm. (**K**) Representative images of SmoA1 in WT and *B9D2^–/–^* cells from live-cell imaging showing the stability of cilia in *B9D2^–/–^* cells is comparable to WT cells without serum stimulation. Scale bar: 10 μm. Data are presented as mean ± SD. **P < 0.01, ***P < 0.001, ****P < 0.0001 by 1-way ANOVA with Dunnett’s test (**C**, **E**, and **F**), by 2-tailed unpaired Student’s *t* test (**I**).

In mammalian cells, TZ integrity is crucial for the proper accumulation of ciliary membrane proteins. Previous studies in *Chlamydomonas reinhardtii* demonstrated that TZ defects result in the abnormal distribution of both membrane and nonmembrane proteins in cilia (22). This result suggests that mislocalization of soluble proteins might occur in TZ-defective mammalian cilia as well. Histone deacetylase 6 (HDAC6) is an enzyme that catalyzes α-tubulin deacetylation during ciliary disassembly (37). We hypothesized that, in the absence of an intact TZ, HDAC6 might enter steady-state cilia and reduce microtubule acetylation. To test this, we stably expressed FLAG-tagged HDAC6 in WT, *B9D2*-KO, and *TMEM67*-KO cells (Figure 3, A and B). Immunostaining for FLAG revealed that HDAC6 was absent from WT cilia but was found in the cilia of *B9D2*-KO and *TMEM67*-KO cells (Figure 3, A and C), though the percentage of cilia with HDAC6 was relatively low (Figure 3C). Further, we stably expressed GFP-FLAG-tagged TTLL5, a glutamylase at the centrosome and cilia (38), in WT and *B9D2*-KO cells (Supplemental Figure 6, A and B). Disrupted distribution of the ciliary TTLL5 was also observed in *B9D2*-KO cells, which had a defective TZ (Supplemental Figure 6, A, C, and D). Interestingly, whereas HDAC6 abnormally accumulated in cilia, the amount of TTLL5 was markedly reduced. These results indicate that defects in the TZ broadly disrupt the bidirectional trafficking of soluble ciliary proteins. Reduced microtubule acetylation has been linked to decreased axonemal stability, which accelerates ciliary disassembly (37, 39–42). To test this, we performed a serum re-addition experiment to monitor ciliary stability during disassembly (Figure 3D). After 6 hours of serum stimulation, 77.8% of WT cells had cilia compared with 41.5% in *B9D2*-KO cells (Figure 3E). In addition, the length of residual cilia in *B9D2*-KO cells was relatively shorter than in WT cells (Figure 3F). We treated the WT, *B9D2*-KO, and *TMEM67*-KO cells with the HDAC6 inhibitor tubacin. Upon tubacin treatment, both global tubulin acetylation and ciliary axonemal microtubule acetylation were increased in *B9D2*-KO and *TMEM67*-KO cells (Figure 3, G–I), whereas levels of ciliary axonemal microtubule acetylation remained comparable in WT cells (Supplemental Figure 6, E–G), suggesting a functional role for HDAC6 in the cilia of *B9D2*-KO and *TMEM67*-KO cells. We also depleted *HDAC6* in *B9D2*-KO cells and *TMEM67*-KO cells using siRNA and observed consistent results with tubacin treatment (Supplemental Figure 6, H–K). These results suggest that the lower stability of cilia in *B9D2*-KO cells may contribute to increased ciliary disassembly, accounting for the reduced ciliation rate observed in these cells. To assess whether ciliary disassembly also occurred more frequently in *B9D2*-KO cells in normal conditions, we conducted live-cell imaging to monitor cilia in both WT and *B9D2*-KO cells by stably expressing a ciliary marker, SMO A1-GFP, which encodes a constitutively active SMO mutant enriched in cilia (Figure 3J). Although the TZ defects in *B9D2*-KO cells resulted in lower concentrations of SMO A1-GFP and photo-bleaching effects diminished its visibility, time-lapse imaging indicated that the presence of cilia in *B9D2*-KO cells was comparable to that in WT cells (Figure 3K). In summary, these findings indicate that the B9-TMEM67 complex functions to exclude cytosolic enzymes from cilia, thereby maintaining ciliary stability during environmental changes. However, the reduced axonemal stability in *B9D2*-KO or *TMEM67*-KO cells does not account for their lower ciliation rates.

### The B9 complex is required for the early steps of ciliogenesis.

**Figure 4:**
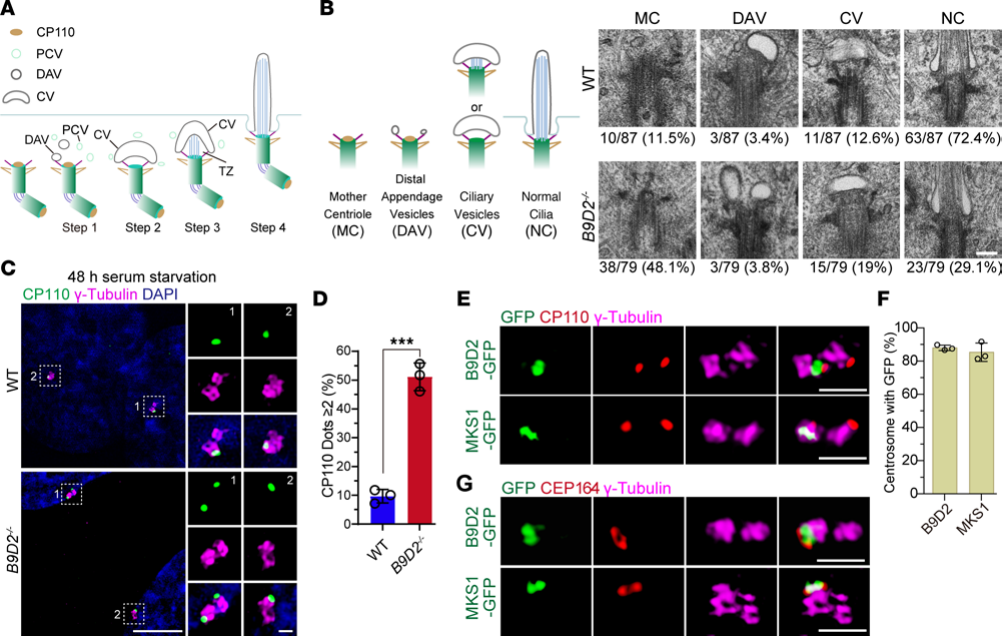
The functions of B9 proteins in early events of ciliogenesis. (**A**) Schematic of intracellular ciliogenesis. PCV, preciliary vesicles; DAV, distal appendage vesicles; CV, ciliary vesicles. (**B**) Quantification of classifications of the mother centriole (basal body) structures in WT and *B9D2^–/–^* cells after 48 hours serum starvation. Scale bar: 200 nm. MC, mother centriole; NC, normal cilia. (**C**) SIM images of CP110 in WT and *B9D2^–/–^* cells after 48 hours of serum starvation. CP110 (green), γ-tubulin (magenta), and DAPI (blue). Scale bars: 5 μm (left) and 1 μm (right). (**D**) Quantification of CP110 localization on the mother and daughter centrioles (2 dots) or only the daughter centriole (1 dot) in **C**. Cells with more than 2 dots were disregarded in the quantification. *n* = 3 replicates. (**E**) SIM images of RPE1 cells stably expressing *B9D2-GFP* and *MKS1-GFP* stained for CP110 (red), γ-tubulin (magenta), and GFP (green). Scale bar: 1 μm. (**F**) Quantification of proliferating RPE1 cells described in **E** showing B9D2 and MKS1 localization on centrioles. *n* = 3 replicates. (**G**) SIM images of RPE1 cells stably expressing *B9D2-GFP* and *MKS1-GFP* stained for CEP164 (red), γ-tubulin (magenta), and GFP (green). Scale bar: 1 μm. Data are presented as mean ± SD. ***P < 0.001 by 2-tailed unpaired Student’s *t* test (**D**).

Ciliogenesis involves multiple steps of cellular processes (Figure 4A) (5, 43). In the intracellular pathway, preciliary vesicles first dock at the distal appendages of the mother centrioles (step 1). The distal appendage–associated vesicles fuse and form the ciliary membrane vesicle, leading to removal of CP110 from the mother centriole (step 2). Subsequently, the TZ is formed by recruiting TZ components, followed by axonemal microtubule extension mediated by intraflagellar transport (step 3). Along with further elongation of the axoneme, the membrane of the ciliary vesicles eventually fuses with the plasma membrane to form a cilium protruding outside the cell surface (step 4). Given that B9 proteins localize to the TZ and their loss induces phenotypes related to TZ dysfunction, it is hypothesized that cells with a disrupted B9 complex fail to assemble an intact TZ (step 3 in Figure 4A). We performed electron microscopy analysis in WT and *B9D2*-KO cells (Figure 4B). Consistent with previous studies, we observed a limited number of abnormal cilia with disrupted TZs. Interestingly, electron microscopy analysis also revealed that mother centrioles in *B9D2*-KO cells frequently lacked associated ciliary vesicles (Figure 4B). Furthermore, immunostaining for CP110 in WT and *B9D2*-KO cells demonstrated that the absence of B9D2 impeded the removal of CP110 from the mother centrioles following serum starvation (Figure 4, C and D). Similar phenotypes were observed in *MKS1*-KO and *B9D1*-KO cells (Supplemental Figure 7, A and B). Previous studies showed that CP110 removal is unaffected in cells without TCTN2 (44), another TZ protein, suggesting different roles for TZ proteins. This finding suggests that B9 proteins are not only essential for TZ assembly but also play a critical role in the early stages of ciliogenesis, which could account for decreased ciliogenesis in mutants of B9 proteins (Supplemental Figure 1, C and D).

Notably, structured illumination microscopy (SIM) analysis showed that both B9D2 and MKS1 were localized to 1 of the 2 centrioles in proliferating cells, most of which do not assemble cilia (Figure 4, E and F). SIM imaging further confirmed that B9D2 and MKS1 localized to the mother centrioles, as evidenced by their proximity to the mother centriole marker CEP164 (Figure 4G). B9 proteins are positioned at the mother centrioles prior to removal of CP110, and their loss diminishes distal appendage–associated vesicles, which suggests that they function in recruitment and/or docking of preciliary vesicles. Thus, these data demonstrate that the loss of B9 subunits disrupts the early steps of ciliogenesis prior to TZ assembly.

### Human variants causing conserved amino acid substitutions in B9D2 are associated with JBTS.

**Figure 5:**
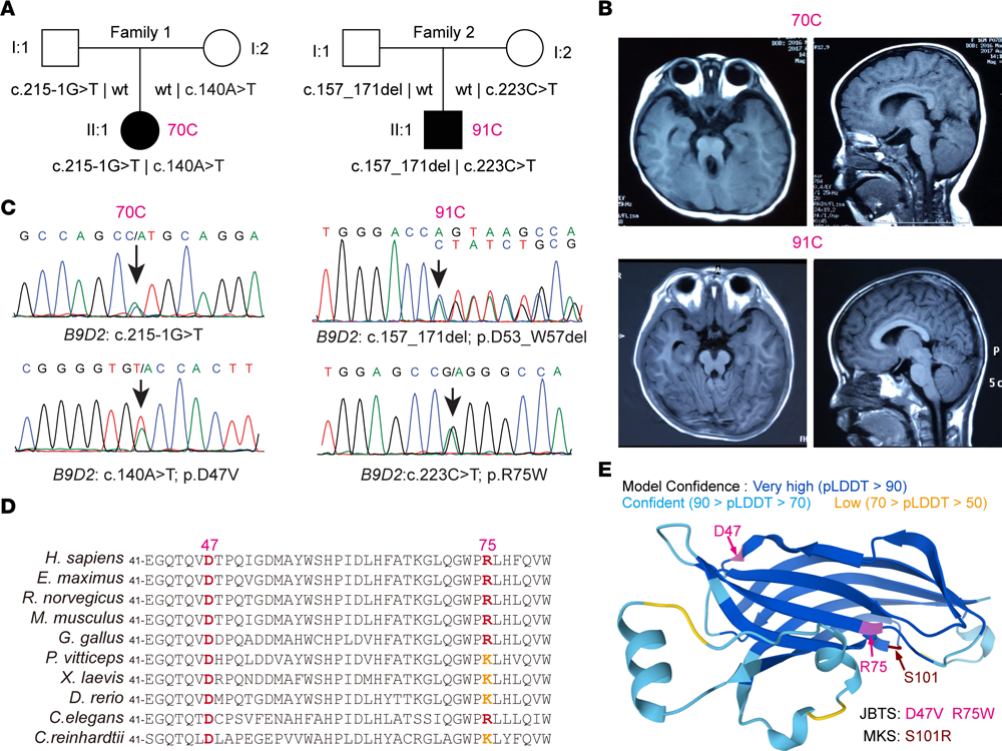
Variants in *B9D2* are associated with JBTS. (**A**) Pedigrees of families with JBTS with *B9D2* variants. (**B**) Representative images of brain MRI for patients 70C and 91C. (**C**) Chromatogram of the genomic DNA sequences showing the *B9D2* variants of the patients. Arrows indicate the variants. (**D**) Alignment of diverse B9D2 sequences reveals that D47 and R75 are evolutionarily conserved. (**E**) Structural interpretation of the pathogenic variants in B9D2. Positions of D47, R75, and S101 are indicated by arrows. The structure was predicted by AlphaFold.

We performed whole-exome sequencing in a cohort of 151 Chinese patients with JBTS and identified 2 affected individuals harboring biallelic variants in *B9D2* (NM_030578.3) (Figure 5A and Supplemental Tables 1 and 2). Brain MRI revealed typical cerebellar vermis hypoplasia in both patients (Figure 5B). The identified variants were c.215-1G>T plus c.140A>T (p.D47V) in patient 70C and c.223C>T (p.R75W) plus c.157_171del (p.D53_W57del) in patient 91C (Figure 5C). Sanger sequencing confirmed that the parents of both individuals were heterozygous carriers (Figure 5C). We applied SpliceAI to predict the splicing impact of the c.215-1G>T variant, which yielded a high score of 0.98, indicating a strong probability of acceptor site loss. RT-PCR followed by Sanger sequencing confirmed 2 aberrant transcripts, resulting in either a frameshift variant (p.W73Vfs*22) or an in-frame deletion of 80 amino acids (p.G72_T151del) in the B9D2 protein (Supplemental Figure 8, A and B). The splicing variant c.215-1G>T in family 1 and the in-frame deletion c.157_171del in family 2 were absent from the control database and dbSNP or gnomAD, while the missense variants c.140A>T in family 1 and c.223C>T in family 2 were found at extremely low allele frequencies in gnomAD (0.0002719 and 0.00007683, respectively), and no homozygotes were recorded. Furthermore, both c.140A>T and c.223C>T were located at conserved positions, with the affected amino acids being conserved across species (Figure 5D). These variants were predicted to be potentially deleterious simultaneously by SIFT (both scores are 0), PolyPhen-2 (0.992 and 0.936), and AlphaMissense (0.93 and 0.657). Thus, in accordance with the American College of Medical Genetics (ACMG) guidelines, these variants were annotated as pathogenic (c.215-1G>T) or likely pathogenic (c.140A>T, c.223C>T and c.157_171del) (45). With advancements in high-accuracy protein structure prediction, disease-associated variants can now be reliably mapped onto atomic structures. The 2 JBTS-relevant residues, Asp47 and Arg75, and a previously reported MKS-relevant residue, Ser101 (20), were highlighted in the B9D2 structure model (Figure 5E). Notably, all 3 highly conserved residues are situated at transition regions between different secondary structures of B9D2 (Figure 5E), suggesting that changes in charge or polarity at these transition regions may disrupt proper conformation of B9D2 and affect its biological function.

### The JBTS-associated variants are functionally distinguished from the MKS-associated variant.

**Figure 6:**
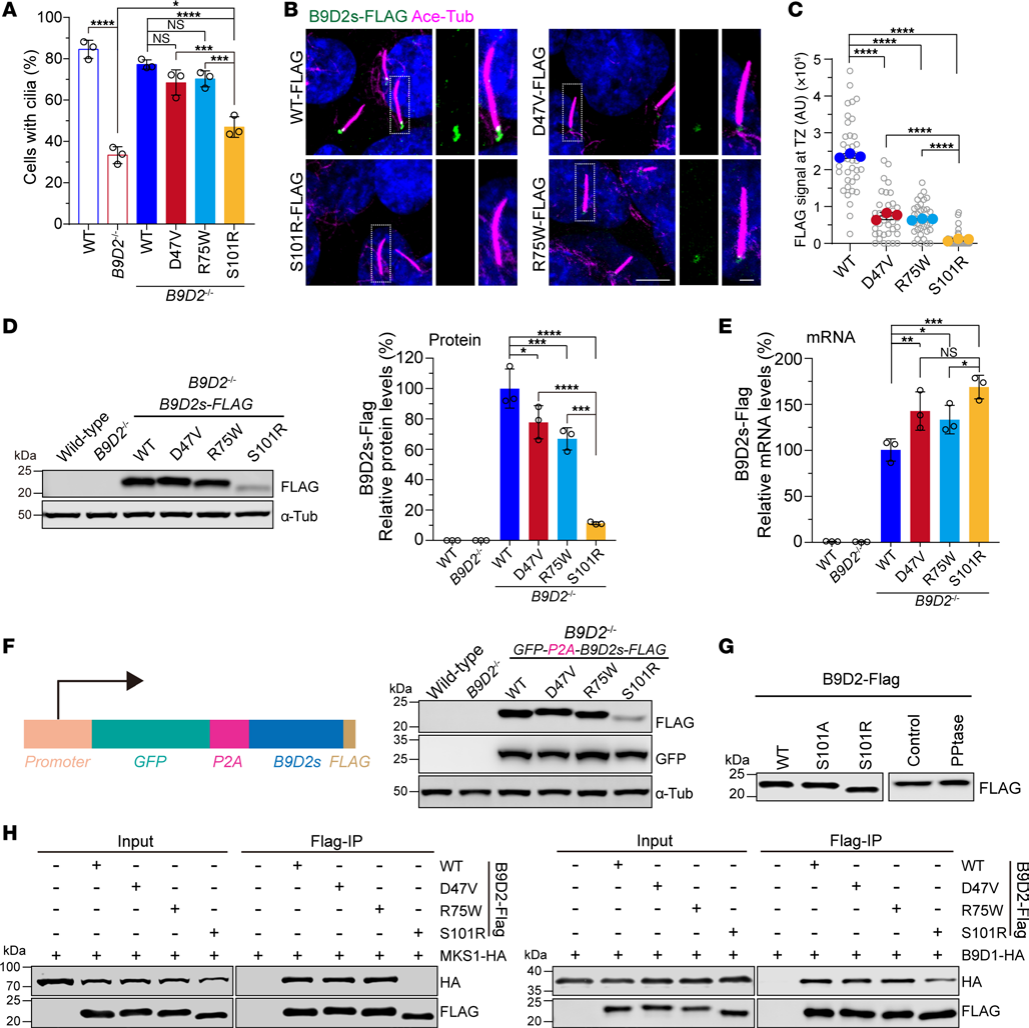
The JBTS- and MKS-associated B9D2 variants compromise TZ localization, but only the MKS variant disrupts the interaction of the B9 complex and affects ciliogenesis. (**A**) Quantification of WT cells, *B9D2^–/–^* cells, and *B9D2^–/–^* cells stably expressing FLAG-tagged *B9D2* variants as indicated with cilia. *n* = 3 replicates. (**B**) Representative images of RPE1 cells stably expressing FLAG-tagged *B9D2* variants stained for FLAG (green), acetylated tubulin (magenta), and DAPI (blue). Scale bars in low or high magnification view are 5 μm or 1 μm, respectively. (**C**) Quantification of fluorescent intensity of FLAG-B9D2 variants at the transition zone in **B** (experiments were done in triplicates). (**D**) Western blots of WT cells, *B9D2^–/–^* cells, and *B9D2^–/–^* cells stably expressing FLAG-tagged *B9D2* variants probed with the indicated antibodies (left panel). Quantification of protein levels of FLAG-B9D2 variants relative to the α-tubulin control (right panel). *n* = 3 replicates. (**E**) Quantification of relative mRNA levels of FLAG-B9D2 variants stably expressed in *B9D2^–/–^* cells. *n* = 3 replicates. (**F**) Schematic of DNA elements of *GFP-P2A-B9D2 variants-FLAG* for the generation of stable *B9D2^–/–^* cell lines expressing *B9D2* variants (left panel). Western blot analysis of WT cells, *B9D2^–/–^* cells, and *B9D2^–/–^* cells stably expressing GFP-P2A-B9D2 variants FLAG probed with the indicated antibodies (right panel). (**G**) Western blot analysis of cells expressing FLAG-tagged WT, S101A, and S101R variants (left). Western blot analysis of cells expressing FLAG-tagged WT B9D2, and the cell lysate was treated with phosphatase or not for 30 minutes at 37° as indicated (right). (**H**) Western blot analysis of products of Co-IP assay using protein lysates from HEK293T cells transfected with indicated plasmids to test the interaction between B9D2 variants and MKS1 (left panel)/B9D1 (right panel). Data are presented as mean ± SD. *P < 0.05, **P < 0.01, ***P < 0.001, ****P < 0.0001; ns, not significant; by 1-way ANOVA with Dunnett’s test (**A**, **C**, **D**, and **E**).

To elucidate the mechanisms by which variants in *B9D2* contribute to ciliary defects and related clinical abnormalities, we investigated the potential pathogenic effects of both JBTS and MKS variants in RPE1 cells. Stable expression of WT and JBTS-associated variants in *B9D2*-KO cells restored ciliogenesis, whereas the MKS-associated variant encoding p.S101R failed to fully rescue the cilia formation defect or induce CP110 removal from the mother centrioles (Figure 6A and Supplemental Figure 9, A and B). Further analysis revealed that in stably expressed cell lines, the enrichment of FLAG-tagged disease-associated variants at the TZ was greatly diminished compared with the control (Figure 6, B and C). In particular, the MKS variant (p.S101R) was nearly undetectable at the TZ in the majority of cells (Figure 6, B and C). To quantify protein levels of the stably expressed FLAG-tagged variants in *B9D2*-KO cells, we conducted immunoblot analyses, which revealed similar protein levels for the JBTS-relevant variants compared with the control; however, the MKS variant, p.S101R, displayed a much lower protein level (Figure 6D). We performed quantitative RT-PCR to evaluate the mRNA levels and found that the mRNA levels of the disease-related variants were comparable to those of the WT mRNA (Figure 6E). To further validate our findings, we generated an additional set of stable cell lines by expressing *B9D2* variants with a GFP tag at the N-terminus, followed by a *P2A* sequence encoding a self-cleaving peptide and a FLAG tag at the C-terminus (Figure 6F). Immunoblot analysis of these lines confirmed a decreased protein level for the MKS variant as well (Figure 6F). These results indicate that the MKS-associated variant, p.S101R, is not stable compared with either WT B9D2 or the JBTS-associated variants. Previous studies showed that the p.S101R variant could be detected at the TZ when it was overexpressed (20). We overexpressed the p.S101R variant in WT RPE1 cells and occasionally observed its localization at the ciliary base, consistent with a previous report (Supplemental Figure 9C). Thus, the mutation in the p.S101R variant does not affect its docking at the TZ but only affects its protein stability. Interestingly, SDS-PAGE analysis showed that the p.S101R variant exhibited a smaller molecular weight (Figure 6, D and F). Given that serine can undergo phosphorylation modifications that would induce a molecular weight shift on SDS-PAGE, we hypothesized that Ser101 might be phosphorylated. To test this, we expressed WT, S101A, and S101R variants in HEK293T cells. Immunoblot analysis indicated that only the S101R variant exhibited a smaller molecular weight, while the S101A variant showed a molecular weight similar to that of the WT protein (Figure 6G). Consistently, phosphatase treatment failed to induce a molecular weight shift of B9D2 as well (Figure 6G). These results suggest that the smaller molecular weight of S101R is not attributed to phosphorylation at Ser101 as opposed to conformation changes. Interestingly, we also observed that transient transfection-induced overexpression resulted in comparable amounts of the WT and S101R variants (Figure 6G).

MKS1, B9D1, and B9D2 form a B9 protein complex. It was reported that the MKS-associated B9D2 variant could not bind MKS1 to form a B9 protein complex (20). We attempted to examine whether the JBTS-associated B9D2 variants could form the B9 complex in comparison with the MKS-associated B9D2 variant and WT B9D2 by co-IP assay. Unlike the MKS-associated B9D2 variant (p.S101R), the JBTS-associated variants (p.D47V and p.R75W) could bind MKS1, while all the B9D2 variants could interact with B9D1 (Figure 6H). Taken together, these findings indicate that the reduced TZ localization of the JBTS-associated variants, p.D47V and p.R75W, is not due to protein instability or their interactions with other B9 subunits. In contrast, the MKS-associated variant, p.S101R, is unstable and cannot form an intact B9 complex for its localization at the TZ.

**Figure 7:**
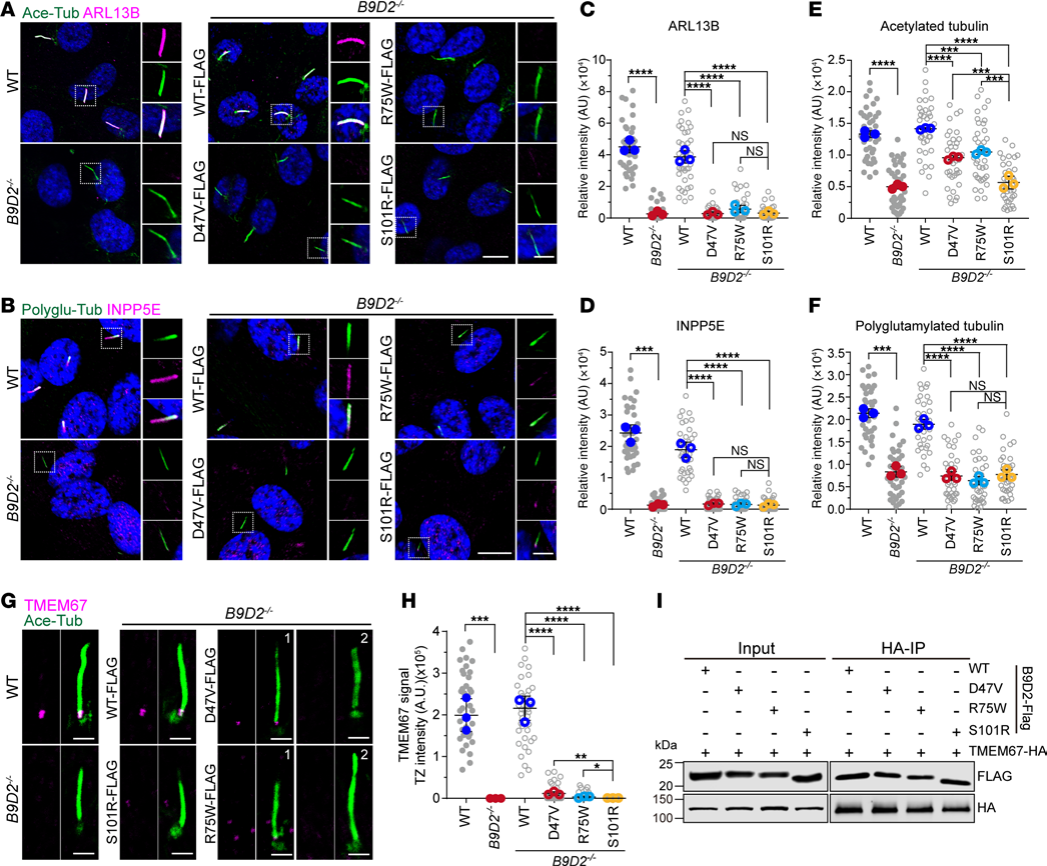
Ciliopathy-associated variants in *B9D2* impair the integrity of cilia. (**A** and **B**) Representative images of WT cells, *B9D2^–/–^* cells, and *B9D2^–/–^* cells stably expressing FLAG-tagged *B9D2* variants stained for acetylated tubulin (green) and ARL13B (magenta) in **A** and polyglutamylated tubulin (green) and INPP5E (magenta) in **B**. DNA was visualized by DAPI (blue). Scale bars in low or high magnification view are 10 μm or 3 μm, respectively. (**C** and **D**) Quantification of ciliary fluorescent intensity of ARL13B (**C**) in **A** and INPP5E (**D**) in **B** (experiments were done in triplicates). (**E** and **F**) Quantification of ciliary fluorescent intensity of acetylated tubulin (**E**) in **A** and polyglutamylated tubulin (**F**) in **B** (experiments were done in triplicates). (**G**) Representative images of WT cells, *B9D2^–/–^* cells, and *B9D2^–/–^* cells stably expressing FLAG-tagged *B9D2* variants stained for acetylated tubulin (green) and TMEM67 (magenta). Scale bar: 2 μm. (**H**) Quantification of fluorescent intensity of TMEM67 at the ciliary transition zone of the cells in **G** (experiments were done in triplicates). (**I**) Co-IP assay using protein lysates from HEK293T cells transfected with FLAG-B9D2 variants and TMEM67-HA plasmids to test the interaction between B9D2 variants and TMEM67. Data are presented as mean ± SD. *P < 0.05, **P < 0.01, ***P < 0.001, ****P < 0.0001, ns, not significant, by 1-way ANOVA with Dunnett’s test (**C**–**F** and **H**).

The B9 complex is critical for concentrating various ciliary membrane proteins within cilia. To evaluate the pathogenicity of the disease-associated variants in the regulation of ciliary membrane proteins, we performed immunostaining using antibodies against ARL13B and INPP5E to assess their localization within cilia. Unlike the functional disparities observed between the JBTS- and MKS-associated variants concerning ciliogenesis and B9 complex formation, both ARL13B and INPP5E were unable to concentrate within the cilia of cells harboring the p.D47V, p.R75W, or p.S101R variants (Figure 7, A–D). Reduced acetylation and polyglutamylation of axonemal tubulin were observed in cells harboring either JBTS- or MKS-associated variants (Figure 7, A, C, E, and F). Additionally, we examined the role of the disease-associated variants in regulating TMEM67 localization. An immunostaining assay indicated that TMEM67 predominantly failed to localize to the TZ in cells harboring either the JBTS- or MKS-associated variants, although a weak TMEM67 signal was detected in a few cells with the JBTS variant (Figure 7, G and H). Interestingly, the interactions between TMEM67 and the disease-associated B9D2 variants were comparable to those of the WT protein (Figure 7I), suggesting that the mislocalization of TMEM67 may be due to reduced localization of the disease-associated variants at the TZ.

## Discussion

Over the past 2 decades, the identification of a rapidly increasing number of ciliopathy-associated genes has largely enhanced our understanding of the role of primary cilia in human diseases (12, 13, 16). Concurrently, considerable advancements have been made in elucidating the molecular basis of ciliopathies. Studies have demonstrated that the proteins forming MKS and NPHP modules in the TZ physically and functionally work together to maintain the selective barrier of the TZ (20, 21, 26–29). In this study, we demonstrate that the B9 proteins and TMEM67 function at the TZ to sustain the posttranslational modification of tubulin in axonemal microtubules. We also reveal that B9 proteins localize to the mother centriole to facilitate the initiation of ciliogenesis by promoting the docking of preciliary vesicles to the distal appendages and the subsequent removal of CP110. Furthermore, we identify variants in *B9D2* that lead to JBTS in a screen of a Chinese patient cohort. Finally, our findings also distinguish the functional implications of JBTS-associated variants in *B9D2* from the MKS-associated variant, offering mechanistic insights into the etiologies of both JBTS and MKS.

Given that eukaryotic cilia comprise over 600 proteins, the entry and retention of ciliary proteins, as well as the exclusion of non-ciliary proteins, are tightly controlled (22, 46). Studies in mammals have shown that cilia lacking a functional TZ fail to accumulate key ciliary membrane proteins. Investigations in *Chlamydomonas* and other models indicate that the TZ plays a role in the composition control of both membrane and nonmembrane proteins in cilia. Consistent with these findings in *Chlamydomonas*, our research provides evidence that the mammalian TZ functions as a selective barrier controlling the entry of the tubulin-modifying enzyme HDAC6, a soluble protein, into cilia. Thus, the TZ in mammalian cilia also plays a critical role in regulating soluble proteins into cilia to maintain ciliary homeostasis. Moreover, we found that the B9 complex anchors TMEM67 to the ciliary membrane at the TZ, elucidating mechanisms that govern the assembly of the MKS module. Overall, this study provides insight into the functional and physical establishment of the selective barrier at the TZ.

Several proteins involved in tubulin posttranslational modifications (PTMs) in cilia have been identified; however, the mechanisms regulating the trafficking of these enzymes into and out of cilia are elusive. This study reveals a connection between the posttranslational modifications of ciliary microtubules and the functionality of the ciliary TZ. Changes in tubulin modifications can influence the binding of microtubule-associated proteins, thereby affecting microtubule-related protein trafficking and signaling (8, 47, 48). We observed in human and mouse cells, as well as in zebrafish, that dysfunction of JBTS-associated genes encoding TZ proteins — including MKS1, B9D1, B9D2, and TMEM67 — results in reduced acetylation and polyglutamylation of axonemal microtubules. Acetylation of α-tubulin, a highly conserved modification first identified in the cilia of *Chlamydomonas reinhardtii* (6, 7), leads to structural rearrangements that may enhance microtubule stability, as evidenced by cryoelectron microscopy studies (49). Supporting this notion, the HDAC6-mediated removal of acetylation from axonemal microtubules is important for ciliary disassembly (37, 40, 41). Consistently, we found that reduced acetylation of axonemal microtubules correlates with compromised ciliary stability. Polyglutamylation, another critical PTM of axonemal microtubules, regulates microtubule-dynein interactions, ciliary dynamics, intraflagellar transport, and cilia-related signaling (9, 50). Although the relevance of altered PTMs of axonemal microtubules in MKS and JBTS has begun to be noticed (50), it remains an intriguing question whether these alterations are functionally linked to the occurrence or progression of ciliopathies.

A notable finding of this study is that B9 proteins not only serve as essential components in constructing the ciliary TZ but also facilitate the early stages of ciliogenesis. Although B9 proteins are not strictly required for cilia formation, ciliary vesicles are frequently absent from mother centrioles in *B9D2*-KO cells. Moreover, loss of any member of the B9 complex compromises the removal of CP110 from the mother centriole. Thus, we have revealed a function of TZ proteins in regulating early events of ciliogenesis. Previous research has demonstrated that the B9D2 protein gradually appears and accumulates at the ciliary base during cilia assembly (5), suggesting that the protein levels of B9D2 around the centriole prior to ciliogenesis are much lower than those in the TZ, thereby rendering the centriole B9D2 nearly undetectable compared with its TZ pool. Although we found that exogenously expressed MKS1 and B9D2 localize to the centriole, we cannot exclude the possibility that overexpression may alter their localization. Further studies are needed to elucidate how B9 proteins exert such a function. In line with this, CEP290, another TZ protein, is localized at centriolar satellites and the centrosome and plays a role in cilia assembly (25, 51).

It is intriguing that variants in *B9D2* can lead to either JBTS or MKS, yet the underlying mechanisms are still not completely clear. In our efforts to identify disease-causing genes within a cohort of patients, we have discovered variants in *B9D2* associated with JBTS. We compared these JBTS-associated variants with the variant linked to MKS. Our analysis revealed no substantial differences in the trafficking of ciliary membrane proteins or the modification of axonemal microtubules between the 2 groups, though a very small amount of the JBTS-associated variants can localize to the TZ and partially retain TMEM67 there. However, the MKS-associated variant exhibited severe defects in ciliogenesis compared with JBTS-associated variants. The rate of ciliation may affect cilia-based signaling within a cell population, potentially underlying the differences in pathology between these variants. The JBTS-associated variants exert a hypomorphic effect, whereas the MKS-associated variant exhibits a null-like behavior and appears to represent a severely damaging variant allele, as indicated by protein instability and an inability to form a functional B9 complex, suggesting distinct functional characteristics compared with those associated with JBTS. Future research will be crucial in dissecting the multifaceted mechanisms by which defects of TZ proteins facilitate ciliopathies.

## Methods

### Sex as a biological variable.

Sex was not considered as a biological variable.

### Patients, exome sequencing, and data processing.

Enrollment criterion was a clinical diagnosis of JBTS, which included typical molar tooth sign or cerebellar vermis hypoplasia on MRI, developmental delay, and hypotonia during infancy. Clinical features were obtained from previous medical records, questionnaires, and comprehensive examinations. Genomic DNA was extracted from peripheral blood samples with the QIAamp DNA Blood Mini kit (51104, QIAGEN). The exome was enriched by SureSelect Human All Exon V6 kit (5190, Agilent Technologies, Inc.) and sequenced with the Illumina NovaSeq 6000 platform. Sequence reads were aligned to the human genome reference (GRCh37/hg19) using the Burrows-Wheeler Aligner along with SAMtools. Picard software was employed to remove PCR duplicates. Variations were called by Genome Analysis Toolkit and annotated with Ensembl Variant Effect Predictor. The detailed strategy used for variant filtering has been described previously, and all candidate variants were classified according to the ACMG guidelines (34, 45). The candidate pathogenic variants and their parental origins were validated by Sanger sequencing.

### Cell culture.

HEK293T cells (ATCC) and NIH-3T3 cells (ATCC) were cultured in DMEM (L110J, BasalMedia) supplemented with 10% FBS (FSP500, ExCell Bio) and 100 IU/mL penicillin/streptomycin (SV30010, Hyclone). RPE-1 cells were cultured in DMEM/F12 1:1 mixture (D8437, Sigma-Aldrich) supplemented with 10% FBS and 100 IU/mL penicillin/streptomycin. For the serum starvation, RPE1 cells were serum starved for 48 hours in DMEM/F12 1:1 mixture with 0.5% serum to induce cilia formation.

CRISPR/Cas9 technology was used to generate mutant cell lines. The sequences targeting the genomic loci of interest were as follows: human *B9D2* (5′-ATGGCTGAGGTGCACGTGAT-3′, 5′-GCGGCATGGAAGCTCCTGTC-3′), human *MKS1* (5′-CTGGAGCACTGACACCGGGG-3′, 5′-TGACACCGGGGAGGCAGTGTAT-3′), human *B9D1* (5′-ACTGTCCTCACAGGGTCTGG-3′, 5′-GTCCTCACAGGGTCTGGAGG-3′), human *TMEM67* (5′-AGCTTGACAAGATGTTAGAT-3′, 5′-TAGTCCACATGCATCAAATG-3′), and mouse *B9d2* (5′-GGCCTTCTCGTACACCTGAG-3′, 5′-ACCTGAGAGGAGCTTCCATG-3′). Annealed sgRNA oligos were cloned into Lenti-CRISPR vector.

The following expression vectors were cotransfected with plasmids psPAX2 (Addgene plasmid,12260) and MD2.g (Addgene plasmid,12259) into HEK293T cells using linearized polyethyleneimine (24765-2, Polysciences Inc.) or Lipocat2000 (AQ11668, Aoqing Biotechnology): pLV-B9D2-Flag, pLV-B9D2-D47V-Flag, pLV-B9D2-R75W-Flag, pLV-B9D2-S101R-Flag, pLV-GFP-P2A-B9D2-Flag, pLV-GFP-P2A-B9D2-D47V-Flag, pLV-GFP-P2A-B9D2-R75W-Flag, pLV-GFP-P2A-B9D2-S101R-Flag, pLV-MKS1-Flag, pLV-Hdac6-Flag, and pLV-SMO-A1-GFP, or the Lenti-CRISPR vectors. The medium containing virus was filtered through a 0.45 μm nitrocellulose (NC) membrane and then were used to infect RPE1 cells with 6 μg/mL of polybrene (sc-134220, Santa Cruz Biotechnology). After 48 hours, the virus-containing medium was replaced with fresh complete medium containing 8 μg/mL of puromycin or 30 μg/mL of blasticidin. The selection lasted for 2 weeks.

### Zebrafish experiments.

All zebrafish strains (Tü background) used were purchased from China Zebrafish Resource Center and maintained on a 14-hour light/10-hour dark cycle at 28.5°C. To generate the *b9d2* mutant zebrafish line, CRISPR/Cas9 was used with the following target sequences: 5′-GCCGATCAATGAATCTATAGCGG-3′ and 5′-GGACAGATCATTGGGGCCACCGG-3′. The sgRNA and Cas9 mRNA were synthesized using Ambion’s MEGAshortscript T7 transcription kit (AM1354) and Ambion’s mMESSAGE mMACHINE T3 transcription kit (AM1348). Coinjection of the 2 sgRNAs led to the deletion between these 2 sites; 50 pg sgRNAs and 100 pg Cas9 mRNA were injected into 1 WT embryo at the 1-cell stage.

### Co-IP assays, mass spectrometry analysis, and Western blot analysis.

For Co-IP assays, HEK293T cells transfected with plasmids were washed by ice-chilled PBS and harvested by scraping in IP buffer (50 mM Tris-HCl, pH 7.5; 150 mM NaCl; 1 mM EDTA; 1 mM NaF; 200 μM Na_3_VO_4_; 5% glycerol; and 0.5% Triton X-100) supplemented with protease inhibitors. Cell lysates were placed on ice for 20 minutes and then cleared by 15,000*g* centrifugation at 4°C for 10 minutes. The supernatant was immunoprecipitated with FLAG M2 beads (A2220, Sigma-Aldrich) or HA beads (A2095, MilliporeSigma) for 3 hours at 4°C. After 3 washes with IP buffer, the proteins on beads were eluted with IP buffer containing 200 μg/mL of Flag or HA peptides. Eluted proteins were digested with trypsin, and the extracted peptides were analyzed using mass spectrometry. B9D1, B9D2, and MKS1, along with 10 high-scoring proteins identified by mass spectrometry, were selected and analyzed using the STRING database (https://string-db.org/). This database integrates extensive interaction data, and the protein-protein associations were visualized through the STRING online tool. For Western blot analysis, cell lysates or eluted IP products were denatured with SDS sample buffer for 10 minutes at 95°C, and then separated by SDS-PAGE. The proteins in the gel were transferred to 0.22 μM NC membranes. After blocking with 5% milk for 30 minutes, the proteins on membrane were probed with the primary and secondary antibodies and detected by fluorescence-based imaging (LI-COR). For quantitative Western blots, infrared dye-labeled secondary antibodies were used to probe the primary antibodies. A LI-COR Odyssey imaging system with Image Studio software was used for the quantification. Briefly, the intensity of the fluorescent signal of each band was normalized by subtracting the background signal. Then, the signal of targeted proteins was normalized to the signal of loading controls. Finally, the fold-changes were obtained by calculating the ratio of the experimental sample to the control. Three biological replicates were used for quantification.

### RNA extraction and real-time qPCR analysis.

Total RNA was extracted from RPE-1 cells using TRIzol reagent (15596018, Life Technologies). cDNAs were generated with HiScript II Q RT SuperMix (R223-01, Vazyme). RT-qPCR reactions were performed with the qTower 3 Real-Time PCR Thermal Cyclers (Analytik Jena) using SYBR Green Premix Pro Taq HS qPCR kit (AG11720, Accurate Biotechnology). Three biological repeats were used in the reactions, and each reaction was run in triplicate using Applied Biosystems QuantStudio 3 or 5 Real-Time PCR Systems. Statistical significance was determined using a 2-tailed *t* test. The primers used in this study were as follows: B9D2-FLAG (F, 5′-GCCAAACGCAAGTGGACACCC-3′; R, 5′- GGACCACACCTGGAAATGGAGC-3′) and GAPDH (F, 5′- CATGAGAAGTATGACAACAGCCT-3′; R, 5′- AGTCCTTCCACGATACCAAAGT-3′).

### Immunofluorescence, imaging, and live-cell imaging.

Cells were cultured on coverslips for the following immunofluorescence experiments. To induce ciliogenesis, cells at 80%–90% confluency were placed in medium with 0.5% serum for 48 hours. Cells were washed with PBS and fixed in 4% paraformaldehyde for 10 minutes, and the fixed cells were permeabilized by ice cold-methanol for 10 minutes. After washing with PBS, cells were incubated in primary antibodies in blocking buffer (PBS, 1% BSA, 0.1% Triton X-100) for 1 hour at room temperature. After washing with PBS, cells were incubated with secondary antibodies in blocking buffer for 1 hour at room temperature. DNA was visualized by DAPI (Sigma-Aldrich). Images of cells were captured using an FV3000 confocal microscope with a 40×/NA1.4 oil objective lens (Olympus). For SIM, images were captured using a HIS-SIM microscope with a 100×/NA1.4 oil objective lens (CSR Biotech). For live-cell imaging, cells were cultured in 4-chamber glass-bottom dishes (J40204, JingAn Biological). Time-lapse microscopy imaging was performed on an Olympus inverted microscope IX83 with a 40×/NA1.3 oil objective lens. Images were acquired every 3 minutes.

### Transmission electron microscopy.

For transmission electron microscopy, cells were cultured in glass-bottom MatTek dishes (P35G-1.5-20-C). After washing with phosphate buffer, cells were fixed in 2.5% glutaraldehyde at room temperature for 2 hours. Then, fixed cells were washed twice with phosphate buffer. The samples were rinsed and post-fixed with 1% OsO_4_ in phosphate buffer at room temperature for 2 hours. After dehydration in a graded series of ethanol (10 minutes each), the samples in dishes were embedded in Epon 812 (Shell Chemical) at 37°C for 2 hours. Next, 70 nm sections were cut by a Leica electron microscope UC7 microtome. A Hitachi H-7650 transmission electron microscope was used for specimen observation.

### Whole-mount IHC and imaging.

To visualize cilia, 1 or 3 dpf zebrafish larvae were fixed in 20% DMSO/methanol overnight at 4°C. Whole-mount immunostaining was carried out following standard protocol as previously reported (33). The stained MZ*b9d2^–/–^* embryos were covered with a coverslip and imaged using a Zeiss LSM980 confocal microscope. The stained *b9d2^–/–^* embryos were mounted in 1% low-melting-point agarose and imaged using a Leica TCS-SP8 confocal microscope. The confocal fluorescence intensity of the same group of experiments was consistently controlled.

### Image analysis.

For quantification of fluorescence intensity, deconvolved 2D maximum intensity projection images were analyzed using Fiji software (NIH). Three regions devoid of the objects were measured to determine the background signal, which was then used as the intensity threshold. Regions of interest were drawn around the objects in multichannel mode to locate the structures of interest. Corresponding single-channel images with the defined regions of interest were used for intensity measurements. Signals below the threshold were recorded as zero.

### Antibodies.

The antibodies used for Western blotting and immunofluorescence were as follows: mouse anti-polyglutamylation modification (1:4,000; AdipoGen; AG-20B-0020-C100), mouse anti-acetylated tubulin (1:25,000; Sigma-Aldrich; T7451), mouse anti-FLAG (1:5,000; Sigma-Aldrich; F1804), and mouse anti–α-tubulin (1:50,000; Proteintech; 66031-1-lg). The secondary antibodies were goat anti-mouse IRDye 680RD (1:15,000; LI-COR; 926-68070). The antibodies used for immunofluorescence were as follows: mouse anti-acetylated tubulin (1:5,000; Sigma-Aldrich; T6793), rabbit anti-CEP164 (1:4,000; Proteintech; 22227-1-AP), rabbit anti-ARL13B (1:2,000; Proteintech; 17711-1-AP), rabbit anti-TMEM67 (1:400; Proteintech; 13975-1-AP), rabbit anti-TCTN1 (1:300; Proteintech; 15004-1-AP), rabbit anti-INPP5E (1:200; Proteintech; 17797-1-AP), rabbit anti-CEP290 (1:500; Proteintech; 17797-1-AP), rabbit anti-RPGRIP1L (1:300; Proteintech; 55160-1-AP), mouse anti-polyglutamylation modification (1:2,000; AdipoGen; AG-20B-0020-C100), rabbit anti-INPP5E (1:300; Proteintech; 17797-1-AP), rabbit anti-CP110 (1:2,000; Proteintech; 12780-1-AP), and alpaca anti-GFP tag (1:1,000; Proteintech; gb2AF488).

### Statistics.

All experiments were performed at least 3 times with 3 biological replicates. In the scatterplots, each point or circle represents 1 sample. In the bar plots, each point or circle represents 1 biological replicate. For quantifications, at least 30 cells in 1 group were used in the experiments. GraphPad Prism (version 8) was used for statistical analysis. Group differences were analyzed using 1-way ANOVA, and a 2-tailed Student’s *t* test was employed to compare 2 groups. All data are reported as mean ± SD unless stated otherwise. A *P* value less than 0.05 was considered significant.

### Study approval.

All zebrafish studies were conducted according to standard animal guidelines and approved by the Animal Care Committee of the Ocean University of China (OUC2012316). Human research was approved by the ethical committees of the National Research Institute for Family Planning. The study followed the tenets of the Declaration of Helsinki, and written informed consent was obtained from patients’ guardians.

### Data availability.

Values for all data points in graphs are available in the Supporting Data Values file. The raw sequence data reported in this paper have been deposited in the Genome Sequence Archive of the National Genomics Data Center, China National Center for Bioinformation (GSA-Human: HRA012836), which is publicly accessible (https://ngdc.cncb.ac.cn/gsa-human).

## Author contributions

RH, YL, MJ, HJ, YS, ML, and MC designed and performed the experiments and analyzed the data. QH, XP, SW, ZL, JL, CL, QS, NL, KW, and HC performed the experiments. DM, ZC, HG, and HY analyzed the data. XM, LW, ML, JP, CZ, and MC conceived the idea, analyzed the data, and wrote the manuscript. All authors approved the final manuscript. RH, YL, MJ, HJ, and YS are co–first authors, as they contributed equally to the manuscript. The order of co–first authorship reflects their respective contributions.

## Funding support

- National Natural Science Foundation of China (32322021, 32541034, and 32570808 to MC, 32125015 and W2411026 to CZ, 82203386 to HJ, and 32370813 and 31991191 to JP).
- Non-profit Central Research Institute Fund of National Research Institute for Family Planning (2020GJZ05 and 2022GJZ01 to ML).
- Natural Science Foundation of Jiangsu Province (BK20231351 to LW).
- Key Research and Development Program of Xuzhou (KC23300 to LW).
- Innovative Research Team of High-level Local Universities in Shanghai (SHSMU-ZDCX20211800).
- Priority Academic Program Development of Jiangsu Higher Education Institutions (PAPD).

## Supplementary Material

Supplemental data

Unedited blot and gel images

Supporting data values

## Version 1 10/30/2025

In-Press Preview

## Version 2 01/16/2026

Electronic publication
