# Supplementary material for: Ciliopathy-related B9 protein complex regulates ciliary axonemal microtubule posttranslational modifications and initiation of ciliogenesis
Source: J Clin Invest. 2025 Oct 30;136(2):e196365. doi: 10.1172/JCI196365 (PMC12807484; doi:10.1172/JCI196365)
Supplement: Supplemental data [file jci-136-196365-s104.pdf]

Ciliopathy-related B9 protein complex regulates ciliary axonemal microtubule post-translational modifications and initiation of ciliogenesis

Ruida He,[1] Yan Li,[1] Minjun Jin,[2,3] Huike Jiao,[1] Yue Shen,[4] Qize Han,[2,3] Xilang Pan,[1] Suning Wang,[1] Zaisheng Lin,[1] Jingshi Li,[1] Chao Lu,[4] Dan Meng,[5] Zongfu Cao,[4] Qing Shang,[6] Nan Lv,[6] Kai Wan,[6] Huafang Gao,[4] Xu Ma,[4] Haiyan Yin,[7] Haishuang Chang,[8] Liang Wang,[9] Minna Luo,[4] Junmin Pan,[3,10] Chengtian Zhao,[2,3] Muqing Cao,[1]

[1]International Peace Maternity and Child Health Hospital, Key Laboratory of Cell Differentiation and Apoptosis of Chinese Ministry of Education, Department of Pathophysiology, Shanghai Jiao Tong University School of Medicine, Shanghai, China.

[2]Institute of Evolution and Marine Biodiversity, Ocean University of China, Qingdao, China.

[3]Laboratory for Marine Biology and Biotechnology, Qingdao Marine Science and Technology Center, Qingdao, China.

[4]National Human Genetic Resources Center, National Research Institute for Family Planning, Beijing, China.

[5]Tianjin Key Laboratory of Food and Biotechnology, School of Biotechnology and Food Science, Tianjin University of Commerce, Tianjin, China.

[6]Rehabilitation Center, Children's Hospital Affiliated to Zhengzhou University, Henan Children's Hospital, Zhengzhou Children's Hospital, Zhengzhou, China.

[7]School of Acupuncture and Tuina, Chengdu University of Traditional Chinese Medicine, Chengdu, China.

[8]Shanghai Institute of Precision Medicine, Shanghai Ninth People's Hospital, Shanghai Jiaotong University School of Medicine, Shanghai, China

[9]School of Life Sciences, Jiangsu Normal University, Xuzhou, China.

[10]MOE Key Laboratory of Protein Sciences, Tsinghua-Peking Center for Life Sciences, School of Life Sciences, Tsinghua University, Beijing, China.

Authorship note: RH, YL, MJ, HJ, and YS contributed equally to this work.

Address correspondence to: Minna Luo, National Human Genetic Resources Center, 12 Dahuisi Road, Beijing, 100081, China. Email: lmn43@163.com; Junmin Pan, Tsinghua University, 1 Qinghuayuan, Beijing, 100081, China. Email: panjunmin@tsinghua.edu.cn; Chengtian Zhao, Ocean University of China, 5 Yushan Road, Qingdao, 266003, China. Email: chengtian_zhao@ouc.edu.cn. Or to Muqing Cao, Department of Pathophysiology, Shanghai Jiao Tong University School of Medicine, 280 South Chongqing Road, Shanghai, 200025, China. Email: muqingcao@sjtu.edu.cn (Lead Contact).

Competing Interest Statement: The authors have declared that no conflict of interest exists.

This PDF file includes:

Supplemental Figures 1 to 9

Supplemental Tables 1 to 2

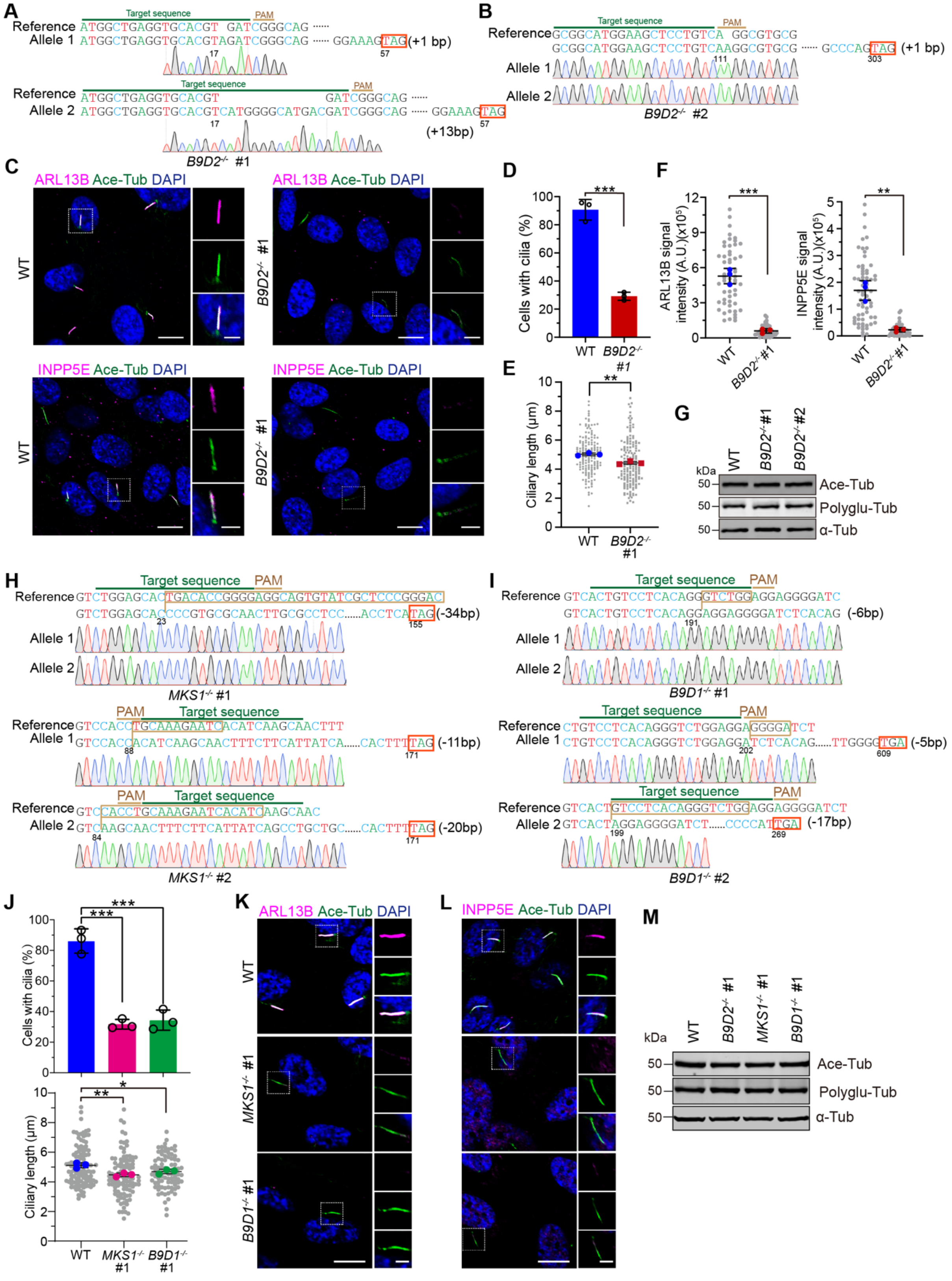

Supplemental Figure 1. Generation of *B9D2*$^{-/-}$, *MKS1*$^{-/-}$, and *B9D1*$^{-/-}$ cell line in RPE1 cells.
(**A** and **B**) The positions of the gRNAs target sites used to generate *B9D2*$^{-/-}$ cell lines and sequences of *B9D2* alleles. (**C**) Representative images of WT and *B9D2*$^{-/-}$ RPE1 cells stained for acetylated tubulin (green), ARL13B (magenta) or INPP5E (magenta), and DAPI (blue). Scale bars in the low or high magnification view are 10 or 3 μm. (**D** and **E**)

Quantification of ciliary number and length in (**C**) (experiments were done in triplicates). (**F**) Quantification of fluorescent intensity of ARL13B and INPP5E in cilia of the cells in (**C**) (experiments were done in triplicates). (**G**) Western blots of WT and *B9D2*$^{-/-}$ RPE1 cells probed with the indicated antibodies. (**H** and **I**) The positions of the gRNAs target sites used to generate *MKS1*$^{-/-}$ and *B9D1*$^{-/-}$ cell lines and sequences of *MKS1*$^{-/-}$ and *B9D1* alleles. (**J**) Quantification of ciliary number and length of WT, *MKS1*$^{-/-}$, and *B9D1*$^{-/-}$ RPE1 cells (experiments were done in triplicates). (**K** and **L**) Representative images of WT, *MKS1*$^{-/-}$, and *B9D1*$^{-/-}$ cells stained for acetylated tubulin (green), ARL13B (magenta), and DAPI (blue) in (**K**), and stained for acetylated tubulin (green), INPP5E (magenta), and DAPI (blue) in (**L**). Scale bars in the low or high magnification view are 10 or 3 μm. (**M**) Western blot analysis of WT, *B9D2*$^{-/-}$, *MKS1*$^{-/-}$, and *B9D1*$^{-/-}$ RPE1 cells probed with the indicated antibodies. Data are presented as mean ± SD. *$P < 0.05$, **$P < 0.01$, ***$P < 0.001$ by 2-tailed unpaired Student's *t* test (**D**, **E**, and **F**), by 1-way ANOVA with Dunnett's test (**J**).

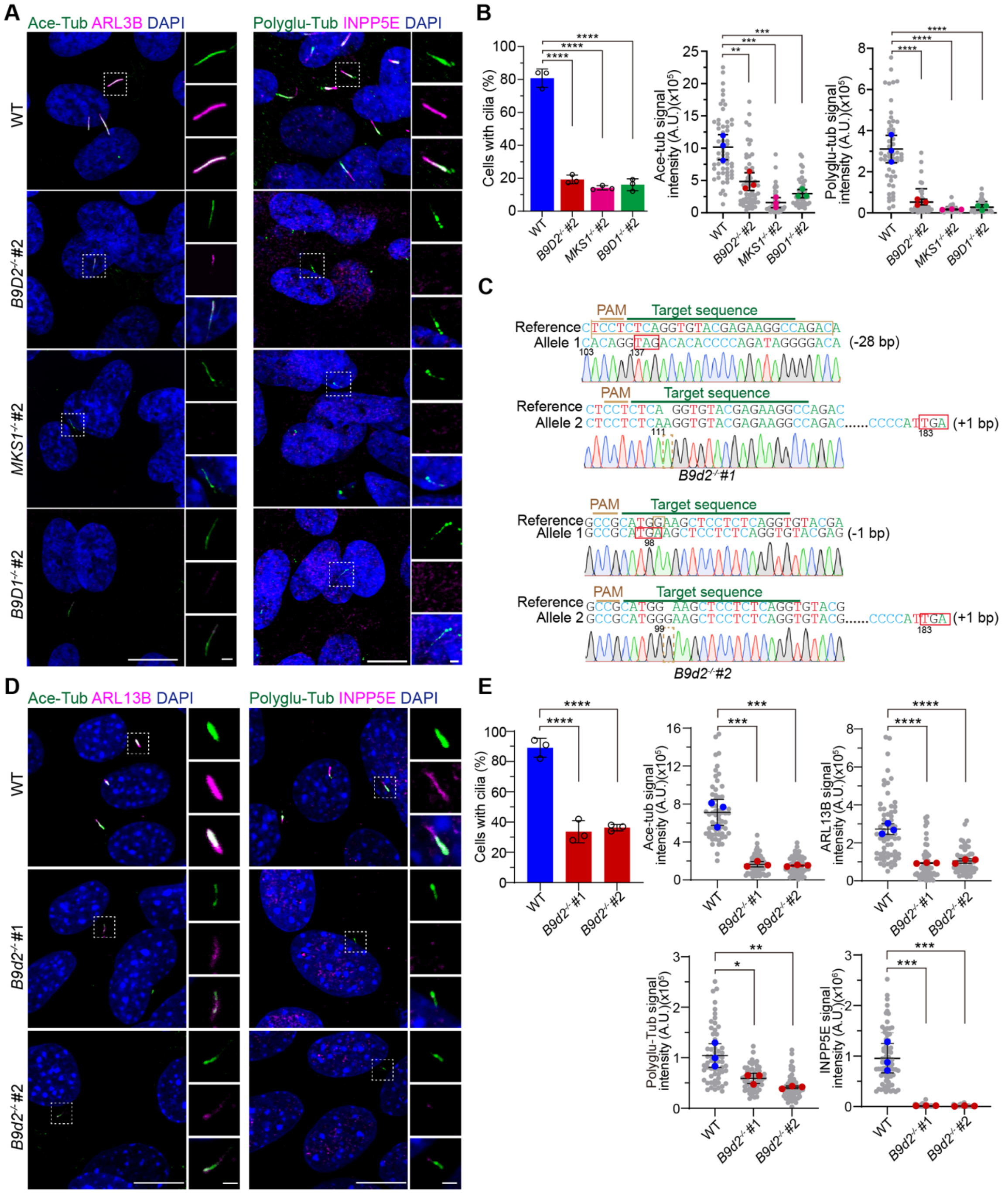

Supplemental Figure 2. Loss of B9 proteins results in altered ciliary composition and defects in ciliogenesis. (**A**) Representative images of WT, *B9D2*$^{-/-}$, *MKS1*$^{-/-}$, and *B9D1*$^{-/-}$ RPE1 cells stained for acetylated tubulin (green), ARL13B (magenta), and DAPI (blue) in the left panel, and stained for polyglutamylated tubulin (green), INPP5E (magenta), and DAPI (blue) in the right panel. Scale bars in the low or high magnification view are 10 or 1 μm. (**B**) Quantification of ciliary number and fluorescent intensity of acetylated tubulin and polyglutamylated tubulin in cilia of the cells in (**A**) (experiments were done in triplicates). (**C**) The positions of the gRNAs target sites used to generate *B9d2*$^{-/-}$ NIH-3T3 cell line and sequences of *B9d2* alleles. (**D**) Representative images of WT and *B9d2*$^{-/-}$ NIH-3T3 cells stained for acetylated tubulin (green), ARL13B (magenta), and DAPI (blue) in the left panel, and stained for polyglutamylated tubulin (green), INPP5E (magenta), and DAPI (blue) in the right panel. Scale bars in the low or high magnification view are 10 or 1 μm. (**E**) Quantification of ciliary number and fluorescent intensity of acetylated tubulin, polyglutamylated tubulin, ARL13B, and INPP5E in cilia of the cells in (**D**) (experiments were done in triplicates). Data are presented as mean ± SD. *$P < 0.05$, **$P < 0.01$, ***$P < 0.001$, ****$P < 0.0001$ by 1-way ANOVA with Dunnett's test (**B** and **E**).

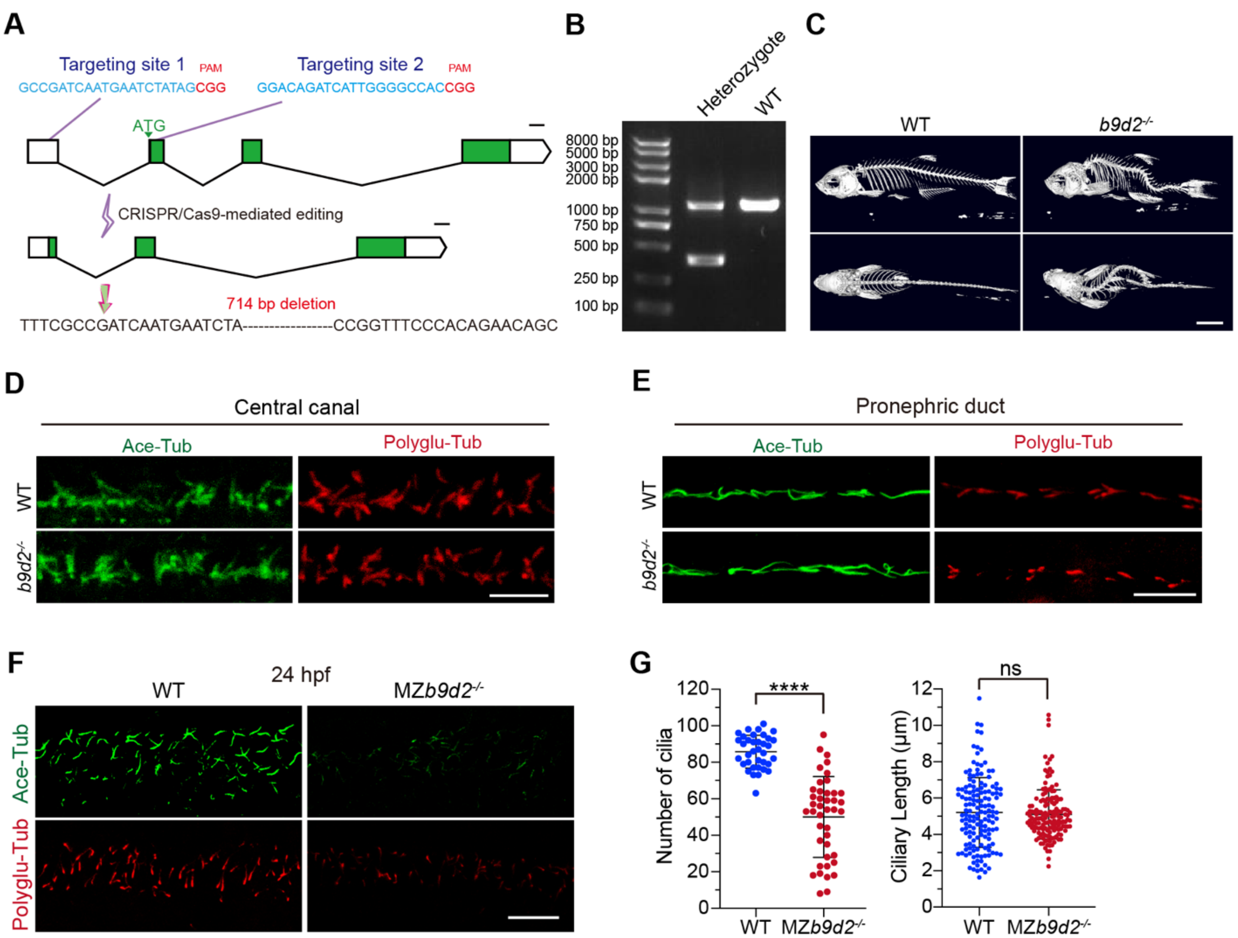

Supplemental Figure 3. Loss of *b9d2* reduces axonemal MT posttranslational modifications in cilia of zebrafish.

(**A**) schematic representation of the genomic structure of WT and *b9d2* mutant alleles generated with CRISPR-Cas9 approach. The target sequences of the two guide RNAs and the PAMs are highlighted. Sanger sequencing indicates that the edited *b9d2* allele causes 714 bp deletion. (**B**) Image of agarose gel electrophoresis of the PCR products from heterozygote and WT. (**C**) Representative micro CT images of WT and *b9d2* mutants at the age of 3 months. Scale bar is 5 mm. (**D**–**E**) Representative confocal images of WT and *b9d2* mutant embryos at 72 hours post fertilization (hpf) stained for acetylated tubulin (green) and polyglutamylated tubulin (red) in the central canal (**D**) and the pronephric duct (**E**). Scale bars are 10 μm. (**F**) Representative confocal images of the central canals in WT and MZ*b9d2* mutant embryos at 24 hpf stained for acetylated tubulin (green) or polyglutamylated tubulin (red). Scale bars are 10 μm. (**G**) Quantification of ciliary number (n = 36 for WT, n = 42 for MZ$b9d2^{-/-}$) and length (n = 150 for WT, n = 150 for MZ$b9d2^{-/-}$) in (**F**). Data are presented as mean ± SD. ns, no significance; ****P < 0.0001 by 2-tailed unpaired Student's t test (**G**).

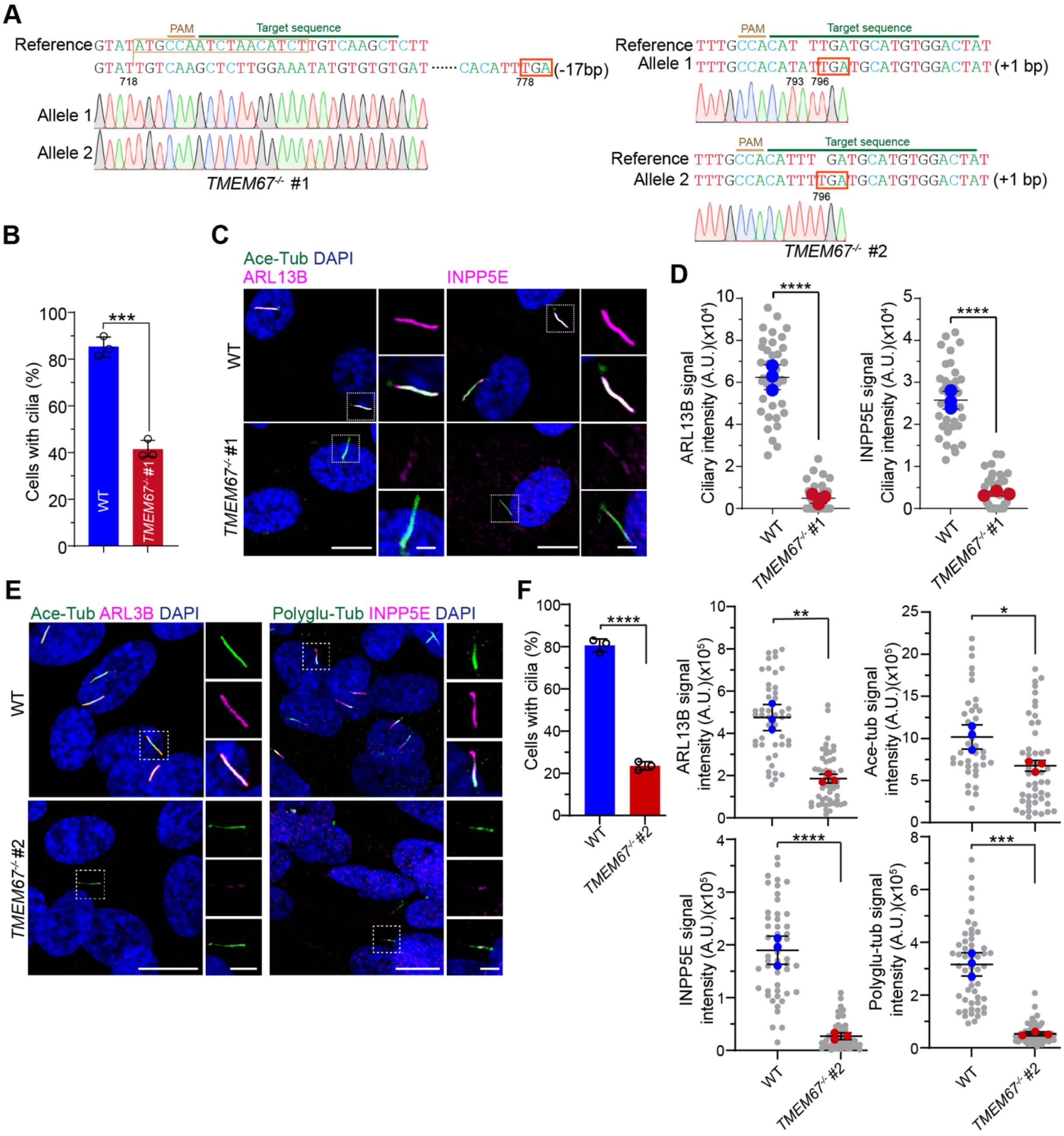

Supplemental Figure 4. Loss of TMEM67 compromises the ciliary accumulation of ARL13B and INPP5E and attenuates posttranslational modifications of the axonemal microtubules.

(**A**) The positions of the gRNAs target site used to generate *TMEM67*$^{-/-}$ RPE1 cell lines and sequences of *TMEM67* alleles. (**B**) Quantification of ciliary number of WT and *TMEM67*$^{-/-}$ cells (*n* = 3 replicates). (**C**) Representative confocal images of WT and *TMEM67*$^{-/-}$ cells stained for acetylated tubulin (green), DAPI (blue), and ARL13B (magenta, left) or INPP5E (magenta, right). Scale bars are 10 or 2 µm. (**D**) Quantification of fluorescent intensity of ARL13B and INPP5E in cilia of the cells in (**C**) (experiments were done in triplicates). (**E**) Representative images of WT and *TMEM67*$^{-/-}$ RPE1 cells stained for acetylated tubulin (green), ARL13B (magenta), and DAPI (blue) in the left panel, and stained for polyglutamylated tubulin (green), INPP5E (magenta), and DAPI (blue) in the right panel. Scale bars in the low or high magnification view are 10 or 2 µm. (**F**) Quantification of ciliary number and fluorescent intensity of acetylated tubulin, polyglutamylated tubulin, ARL13B, and INPP5E in cilia in (**E**) (experiments were done in triplicates). Data are presented as mean ± SD. *P < 0.05, **P < 0.01, ***P < 0.001, ****P < 0.0001 by 2-tailed unpaired Student's *t* test (**D** and **F**).

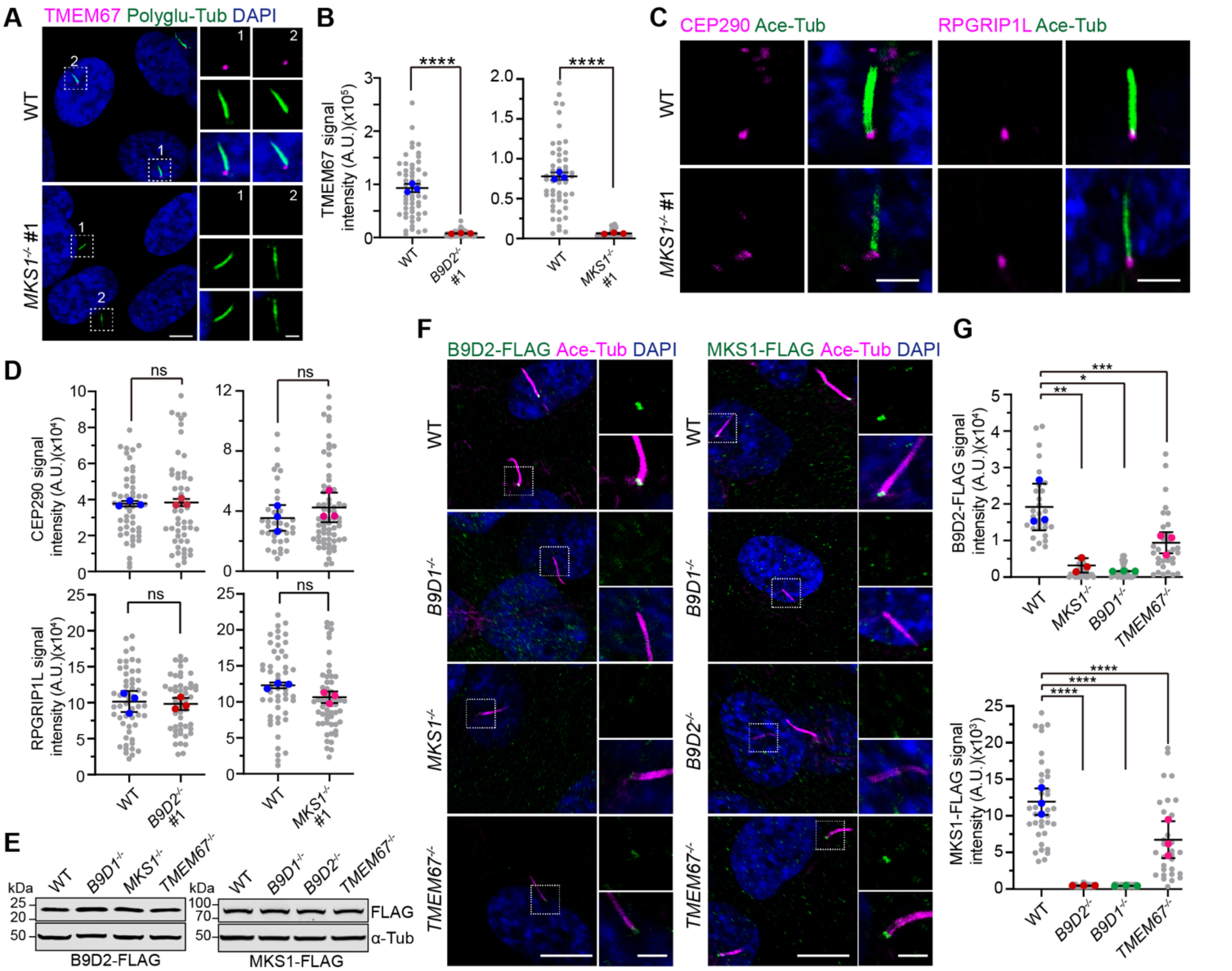

Supplemental Figure 5. The localization of proteins at the transition zone in *B9s*$^{-/-}$ and *TMEM67*$^{-/-}$ RPE1 cells. (**A**) Representative images of WT and *MKS1*$^{-/-}$ cells stained for acetylated tubulin (green), TMEM67 (magenta), and DAPI (blue). Scale bars in the low or high magnification view are 5 or 1 μm. (**B**) Quantification of fluorescent intensity of TMEM67 at the transition zone of the cells in **Figure 2F** and in (**A**) (experiments were done in triplicates). (**C**) Representative images of WT and *MKS1*$^{-/-}$ cells stained for acetylated tubulin (green), CEP290 (red, left), RPGRIP1L (red, right) and DAPI (blue). Scale bars are 2 μm. (**D**) Quantification of fluorescent intensity of CEP290 and RPGRIP1L at the transition zone of the cells in **Figure 2G** (left) and (**C**) (right) (experiments were done in triplicates). (**E**) Western blot analysis of cells probed with the anti-FLAG and anti-α-Tubulin antibodies. *B9D2-FLAG* or *MKS1-FLAG* is stably expressed in the indicated genotypes of RPE1 cells. (**F**) Representative images of the indicated cells stained for FLAG (green), acetylated tubulin (magenta), and DAPI (blue). Scale bars are 10 or 2 μm. (**G**) Quantification of fluorescent intensity of B9D2 and MKS1 at the transition zone of the cells in (**F**) (experiments were done in triplicates). Data are presented as mean ± SD. ns, no significance; **P* < 0.05, ***P* < 0.01, ****P* < 0.001, *****P* < 0.0001 by 2-tailed unpaired Student's *t* test (**B** and **D**), or by 1-way ANOVA with Dunnett's test (**G**).

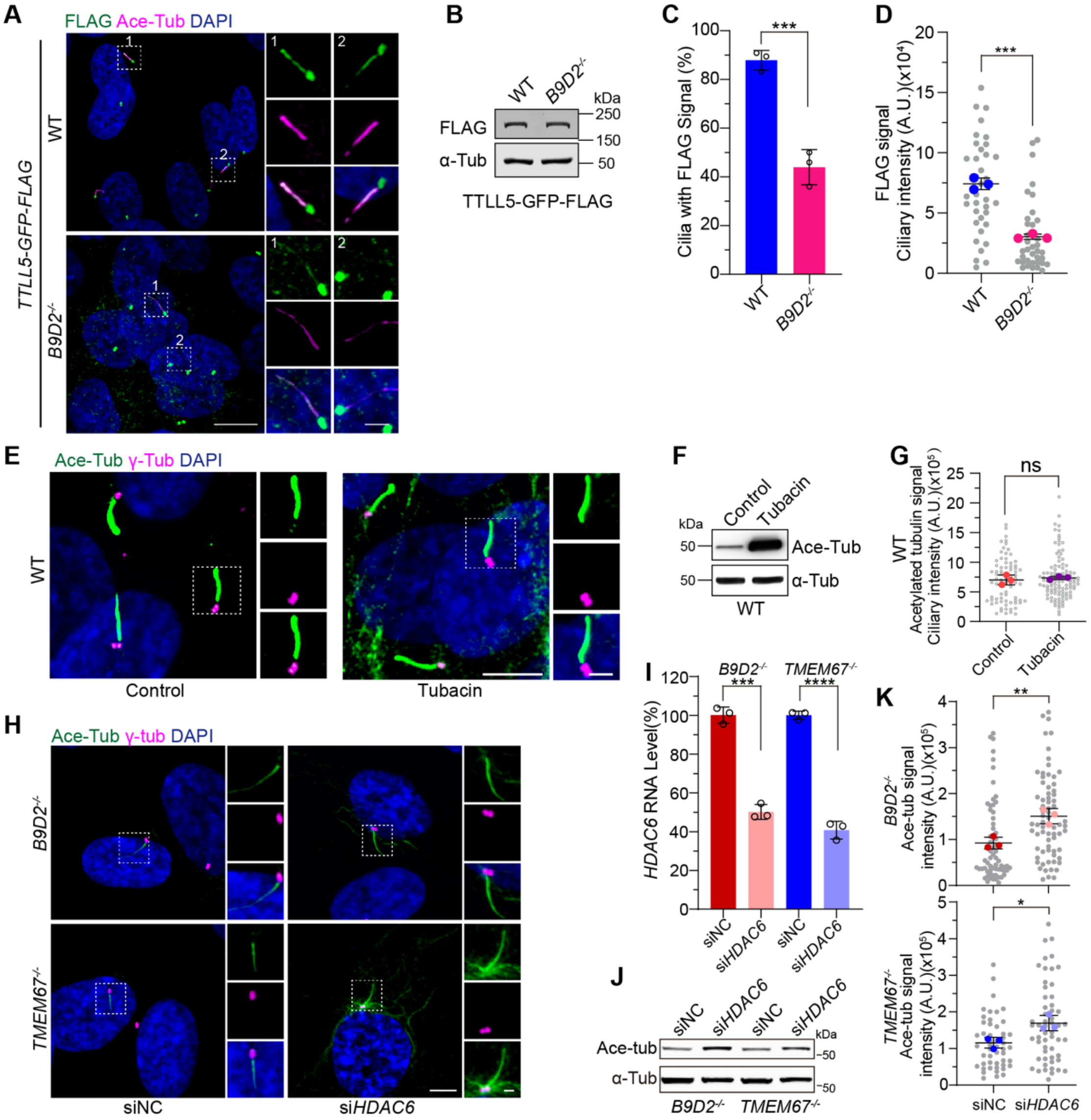

Supplemental Figure 6. Disruption of the B9-TMEM67 complex compromises the TZ selective barrier for soluble proteins.

(**A**) Representative images of WT and *B9D2*$^{-/-}$ RPE1 cells expressing GFP-FLAG-tagged TTLL5 stained for FLAG (green), acetylated tubulin (magenta), and DAPI (blue). Scale bars in the low or high magnification view are 10 or 2 μm. (**B**) Western blots of WT and *B9D2*$^{-/-}$ RPE1 cells expressing GFP-FLAG-tagged TTLL5 probed with antibodies against FLAG and α-Tubulin. (**C**) Quantification of the TTLL5-positive cilia of cells in (**A**) (n = 3 replicates). (**D**) Quantification of fluorescent intensity of TTLL5 in cilia of cells in (**A**) (experiments were done in triplicates). (**E**) Representative images of WT RPE1 cells, treated with or without Tubacin (2 μM) for 24 h, stained for acetylated tubulin (green), γ-tubulin (magenta), and DAPI (blue). Scale bar in the low or high magnification view is 5 μm or 1 μm. (**F**) Western blots of WT RPE1 cells in (**E**), probed with antibodies against acetylated tubulin and α-Tubulin. (**G**) Quantification of fluorescent intensity of acetylated tubulin in cilia in (**E**) (experiments were done in triplicates). (**H**) Representative images of *B9D2*$^{-/-}$ and *TMEM67*$^{-/-}$ cells, treated with siRNAs, stained for acetylated tubulin (green), γ-tubulin (magenta), and DAPI (blue). Scale bar in the low or high magnification view is 5 μm or 1 μm. (**I**) Quantification of relative mRNA levels of *HDAC6* in *B9D2*$^{-/-}$ and *TMEM67*$^{-/-}$ cells in (**H**) (n = 3 replicates). (**J**) Western blots of *B9D2*$^{-/-}$, and *TMEM67*$^{-/-}$ cells in (**H**) probed with antibodies against acetylated tubulin and α-Tubulin. (**K**) Quantification of fluorescent intensity of acetylated tubulin in cilia in (**H**) (experiments were done in triplicates). Data are presented as mean ± SD. ns, no significance; *P < 0.05, **P < 0.01, ***P < 0.001, ****P < 0.0001 by 2-tailed unpaired Student's t test (**C**, **D**, **G**, **I**, and **K**).

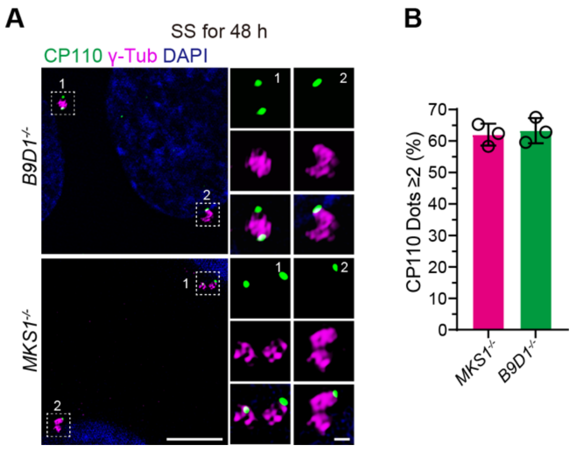

Supplemental Figure 7. The B9 proteins function in CP110 removal from the mother centriole upon ciliogenesis.

(**A**) Representative images of CP110 in *B9D1*$^{-/-}$ and *MKS1*$^{-/-}$ cells after 48 hours serum starvation. CP110 (green), γ-tubulin (magenta), and DAPI (blue). Scale bar in the low or high magnification view is 5 μm or 1 μm. (**B**) Quantification of CP110 localization on both mother and daughter centrioles (two dots) or only the daughter centriole (one dot) in (**A**) (n = 3 replicates).

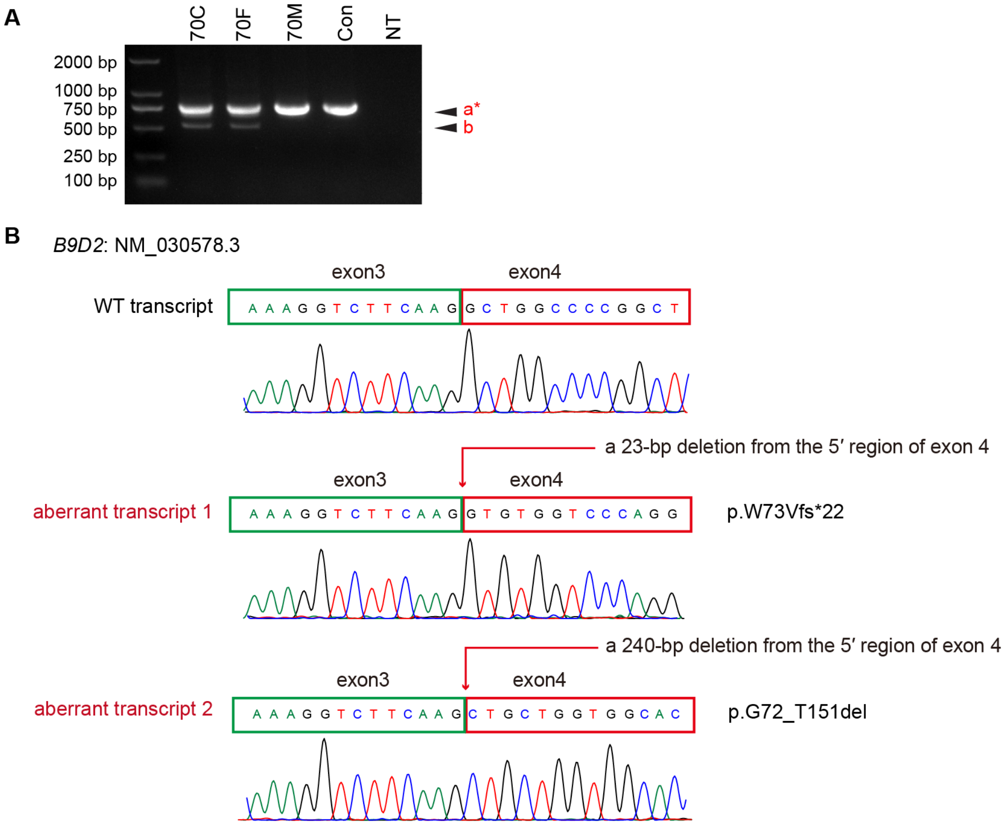

Supplemental Figure 8. Splicing defect caused by the c.215-1G>T variant.
(**A**) Agarose gel electrophoresis of PCR products from the proband (70C), father (70F), mother (70M), healthy control (Con), and no-template control (NT); a* indicates mixed products of the WT transcript (763 bp) and the aberrant transcript 1 (740 bp), and b indicates the product of the aberrant transcript 2 (523 bp). (**B**) Sanger sequencing chromatograms showing aberrant *B9D2* transcripts caused by the c.215-1G>T variant.

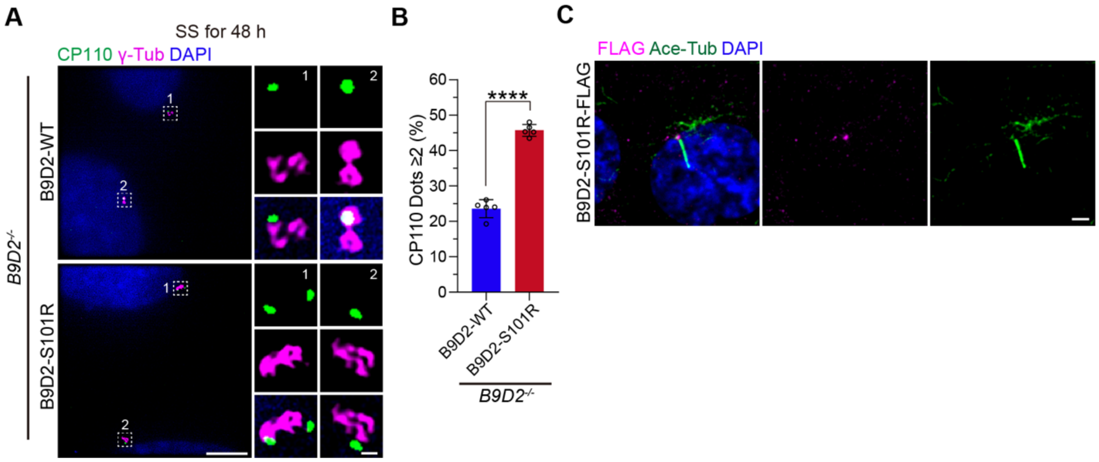

Supplemental Figure 9. The p.S101R variant of B9D2 impairs CP110 removal from the mother centriole. (**A**) Representative images of CP110 in *B9D2*$^{-/-}$ cells expressing WT B9D2 or p.S101R variant after 48 hours serum starvation. CP110 (green), γ-tubulin (magenta), and DAPI (blue). Scale bar in the low or high magnification view is 5 µm or 1 µm. (**B**) Quantification of CP110 localization on both mother and daughter centrioles (two dots) or only the daughter centriole (one dot) in (**A**) (*n* = 5 replicates). (**C**) Representative images of WT RPE1 cells expressing FLAG-tagged B9D2 p.S101R variant stained for FLAG (magenta), acetylated tubulin (green), and DAPI (blue). Scale bar is 2 µm. Cells with more than two CP110 dots were disregarded in the quantification. Data are presented as mean ± SD. ****$P < 0.0001$ by 2-tailed unpaired Student's *t* test (**B**).

Patient ID	70C	91C
Gender	Female	Male
Age	3.5 years	9 years
Ethnicity	Chinese	Chinese
Mutation 1	c.215-1G>T	c.157_171del
		p.D53_W57del
Mutation 2	c.140A>T	c.223C>T
	p.D47V	p.R75W
Molar Tooth Sign	+	+
Developmental delay	+	+
Hypotonia	+	+
Respiratory abnormality	+	+
Oculomotor Apraxia	+	+
Retinal involvement	ND	-
Renal involvement	-	-
Liver involvement	ND	ND
Limb anomalies	-	-

ND, Not Determined.

Supplemental Table 1. Clinical features and genotypes of *B9D2*-related Joubert syndrome patients.

Sample ID	Genomics	Zygosity	Gene	cDNA	Mutation	Existing variation	SIFT	PolyPhen	Alphamissense	AF in gnomAD
70C	chr19:g.41860919C>A	Heterozygous	*B9D2*	NM_030578.3:c.215-1G>T			NA	NA	NA	0
70C	chr19:g.41863876T>A	Heterozygous	*B9D2*	NM_030578.3:c.140A>T	p.D47V	rs770930532	deleterious (0)	probably_damaging(0.992)	pathogenic (0.9298)	0.0002719
70C	chr15:g.90192266C>T	Heterozygous	*KIF7*	NM_198525.2:c.862G>A	p.A288T	rs1308503261	deleterious (0.01)	probably_damaging(0.99)	ambiguous (0.4342)	0.000006786
91C	chr19:g.41860910G>A	Heterozygous	*B9D2*	NM_030578.3:c.223C>T	p.R75W	rs765309540	deleterious (0)	probably_damaging(0.936)	pathogenic (0.657)	0.000004548
91C	chr19:g.41863847_41863861del	Heterozygous	*B9D2*	NM_030578.3:c.157_171del	p.D53_W57del		NA	NA	NA	0

AF: allele frequency

Supplemental Table 2. Rare damaging variants of known JBTS genes in the *B9D2*-related patient.
