# Supplementary material for: Ciliopathy-related B9 protein complex regulates ciliary axonemal microtubule posttranslational modifications and initiation of ciliogenesis
Source: J Clin Invest. 2025 Oct 30;136(2):e196365. doi: 10.1172/JCI196365 (PMC12807484; doi:10.1172/JCI196365)

Full unedited blot for Figure 2 B

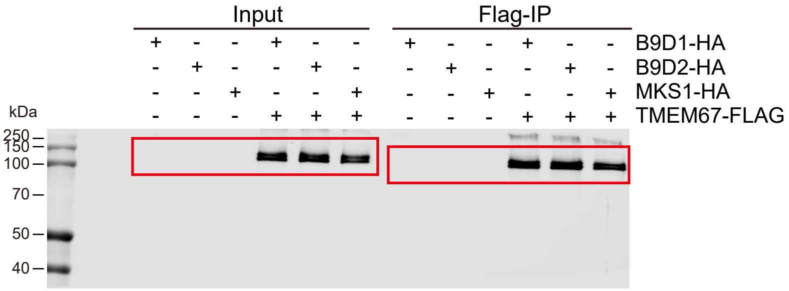

Antibody: FLAG

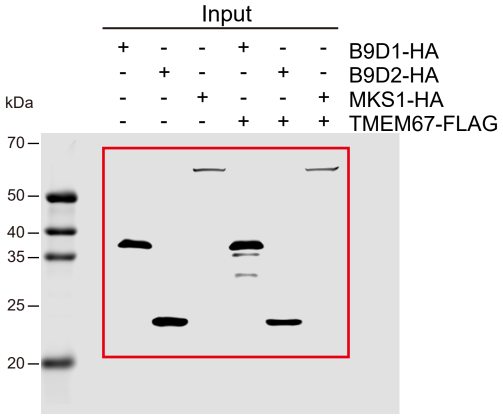

Antibody: HA

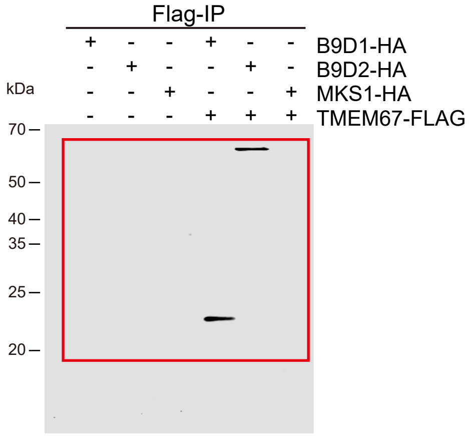

Antibody: HA

Full unedited blot for Figure 3 B

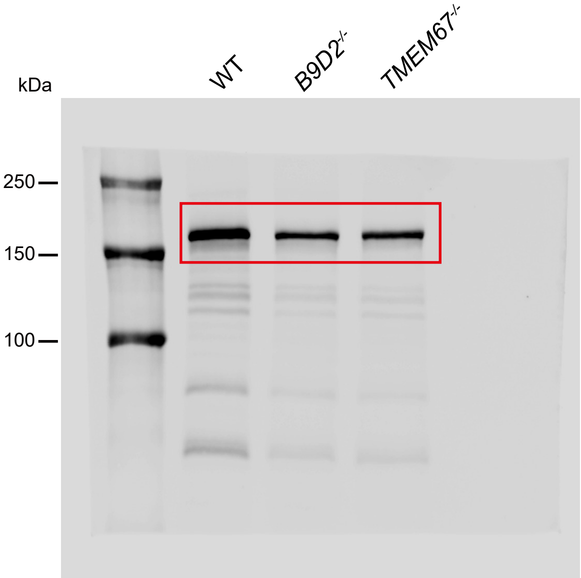

Antibody: FLAG

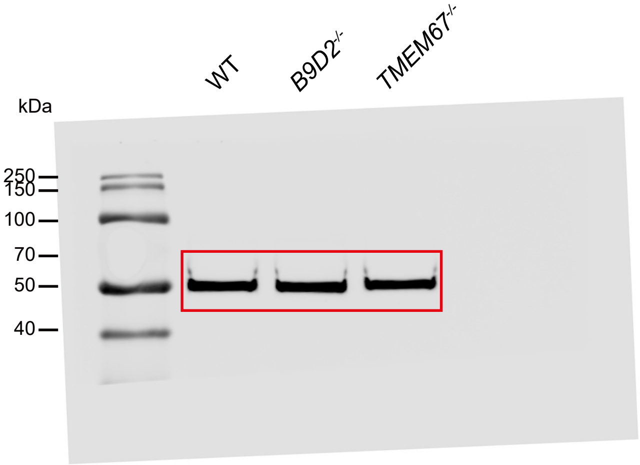

Antibody: α-Tub

Full unedited blot for Figure 3 H

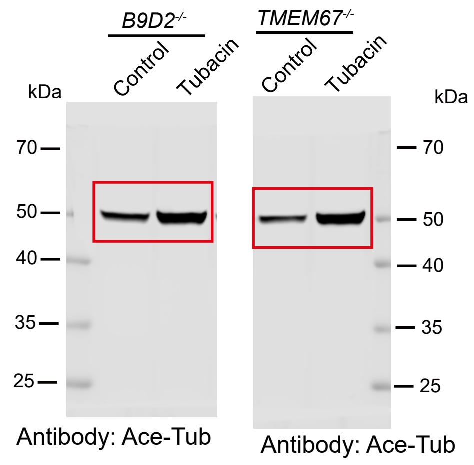

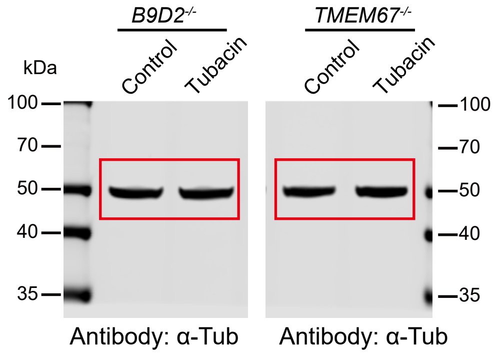

Full unedited blot for Figure 6 D

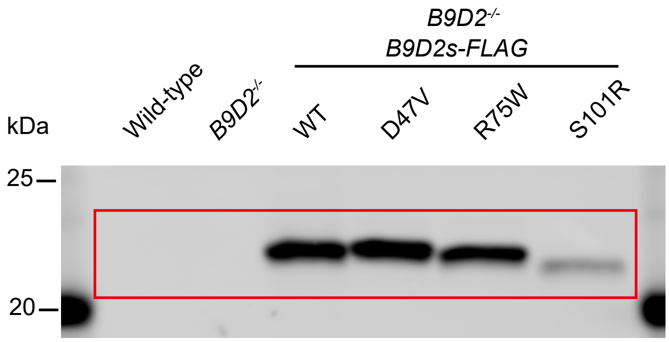

Antibody: FLAG

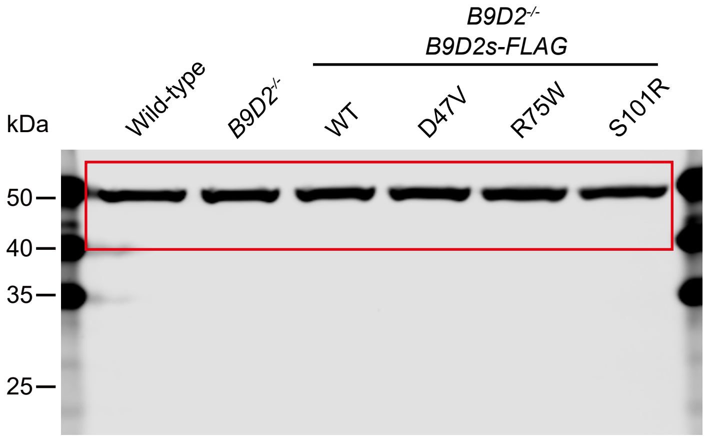

Full unedited blot for Figure 6 F

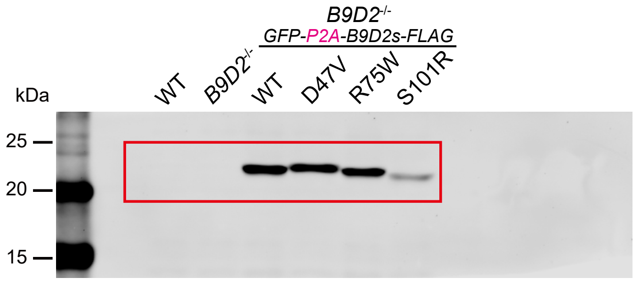

Antibody: FLAG

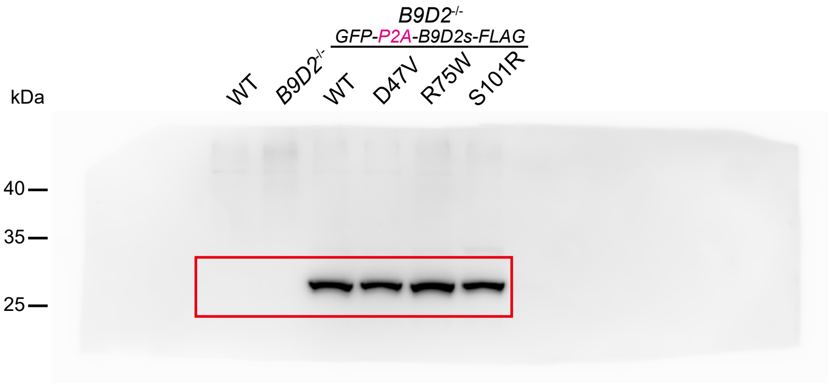

Antibody: GFP

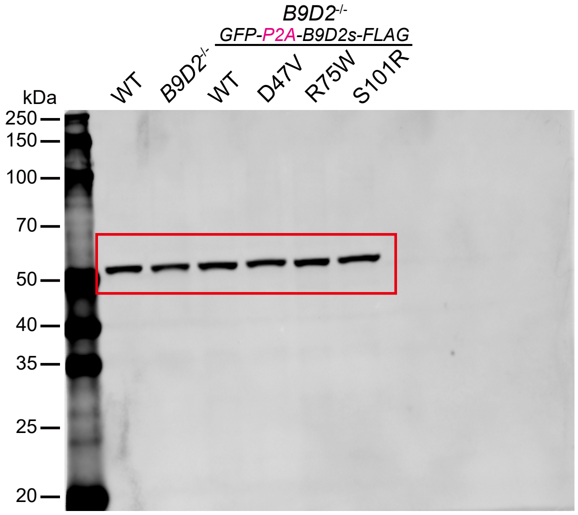

Antibody: α-Tub

Full unedited blot for Figure 6 G

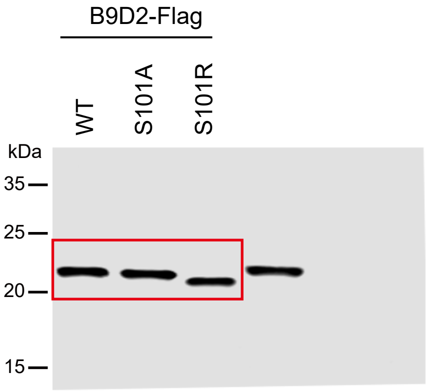

Antibody: FLAG

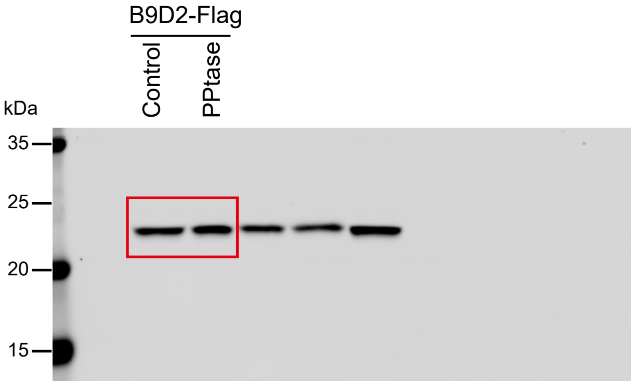

Antibody: FLAG

Full unedited blot for Figure 6 H

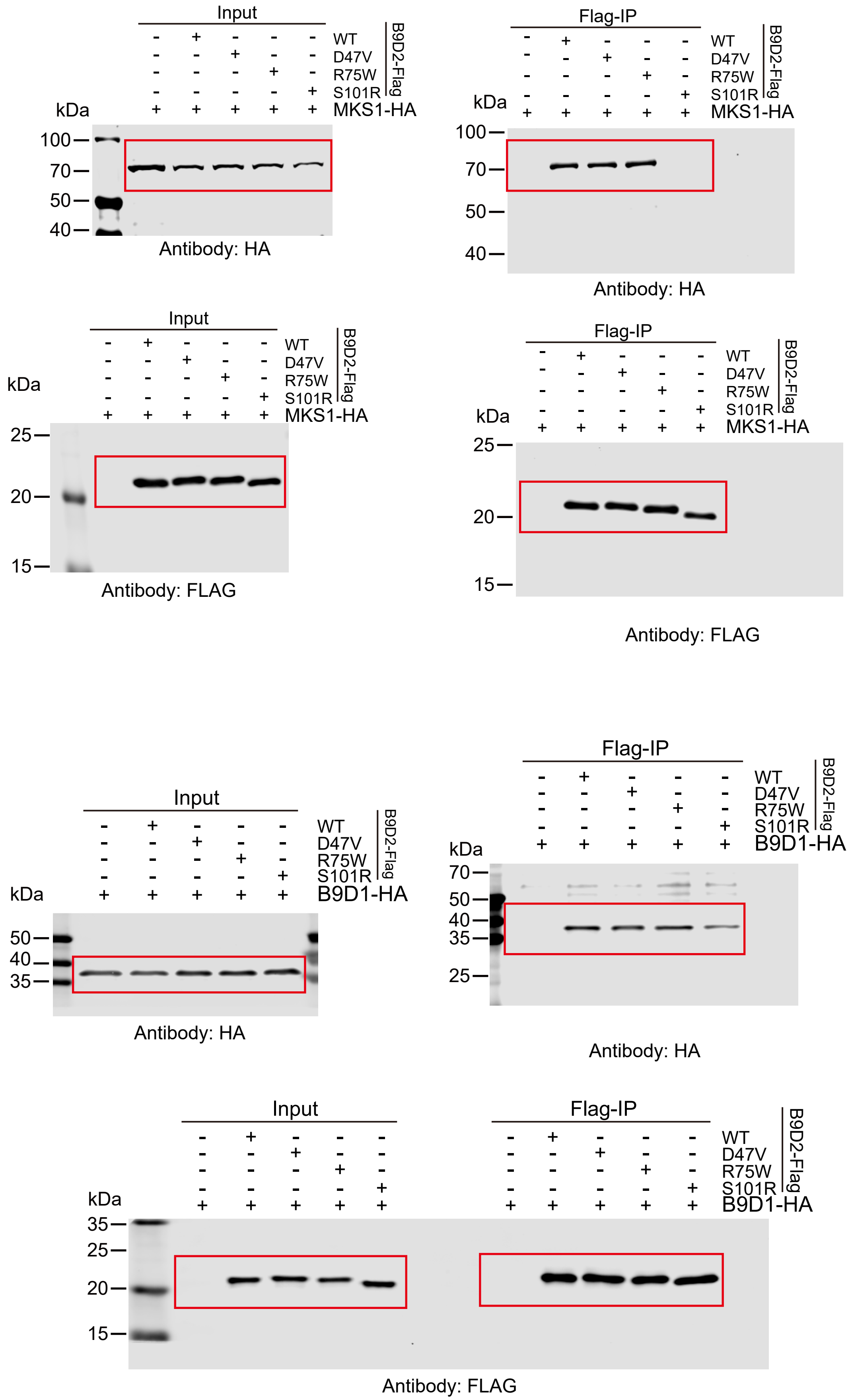

Full unedited blot for Figure 7 I

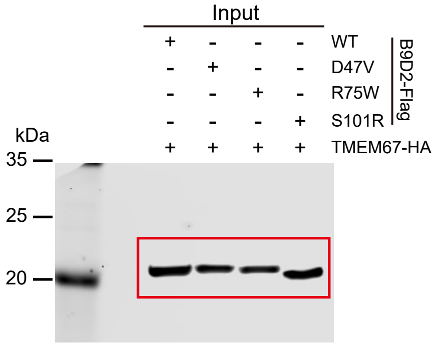

Antibody: FLAG

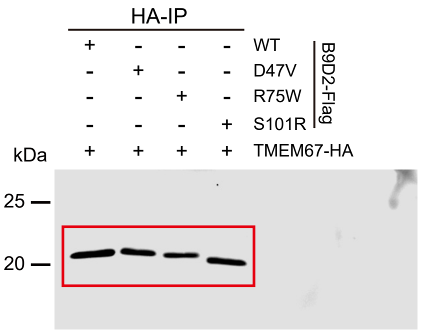

Antibody: FLAG

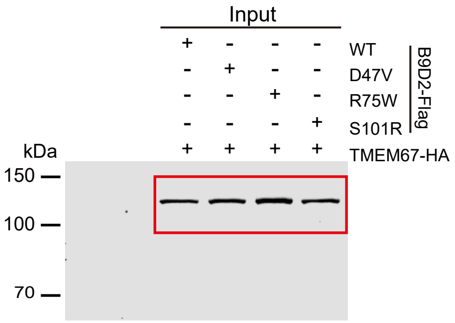

Antibody: HA

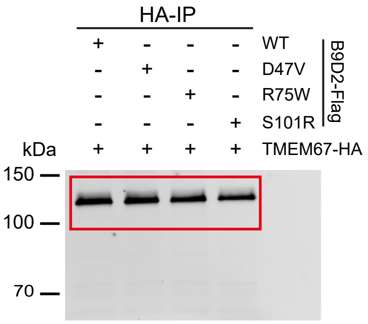

Antibody: HA

Full unedited blot for Supplemental Figure 1 G

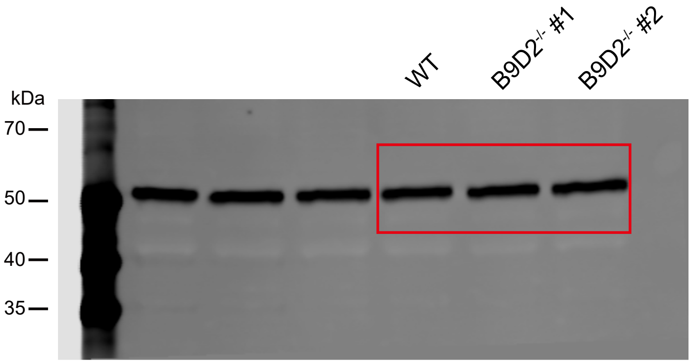

Antibody: Ace-Tub

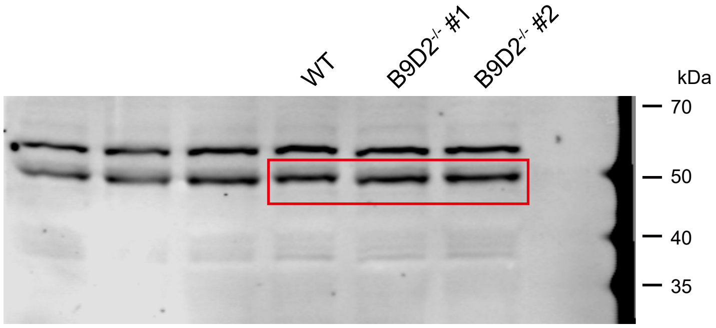

Antibody: Polyglu-Tub

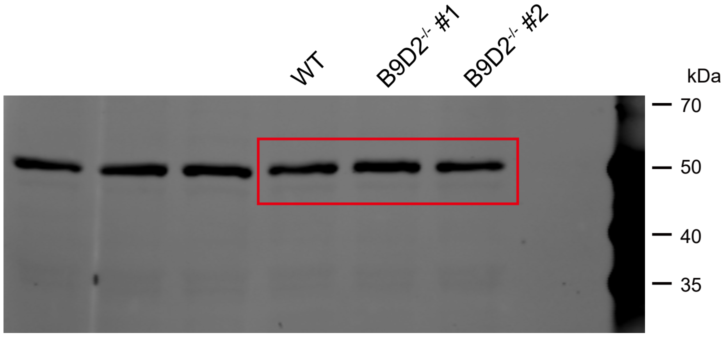

Antibody: α-Tub

Full unedited blot for Supplemental Figure 1 M

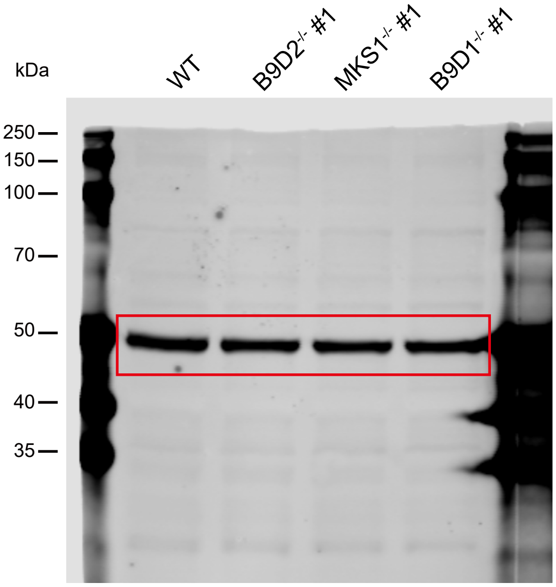

Antibody: Ace-Tub

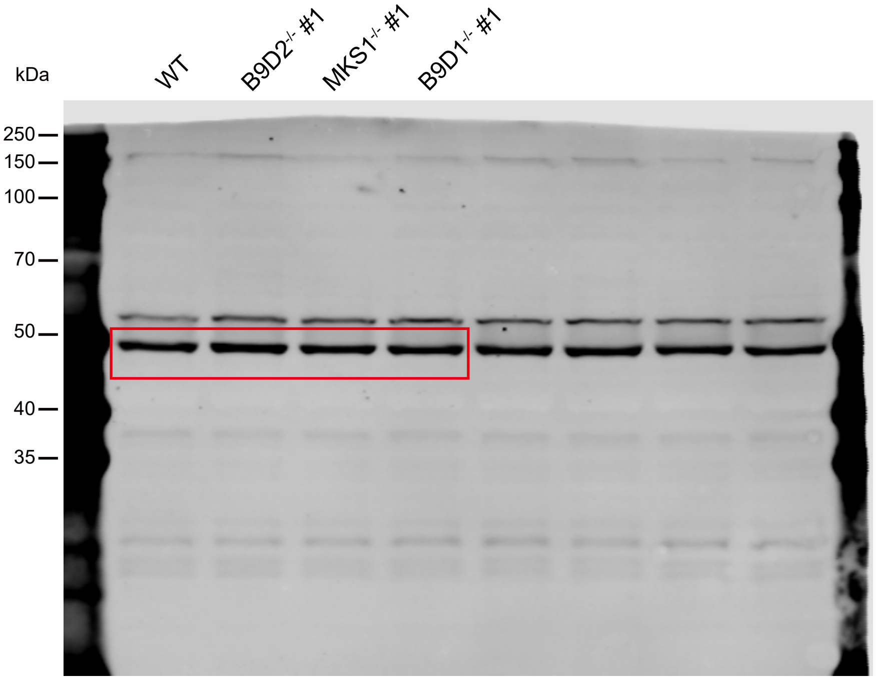

Antibody: Polyglu-Tub

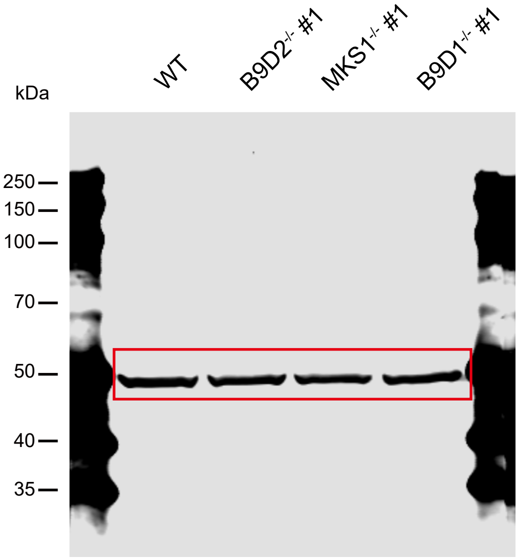

Antibody: α-Tub

Full unedited gel for Supplemental Figure 3 B

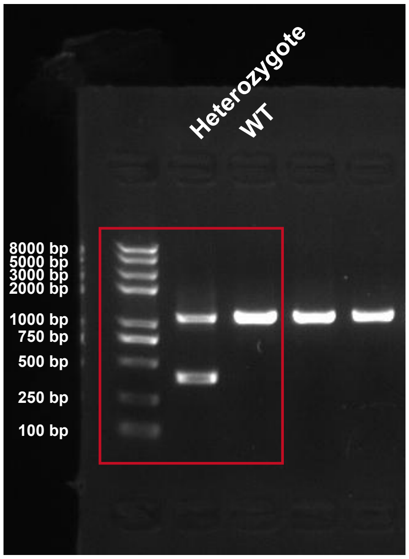

Full unedited blot for Supplemental Figure 5 E

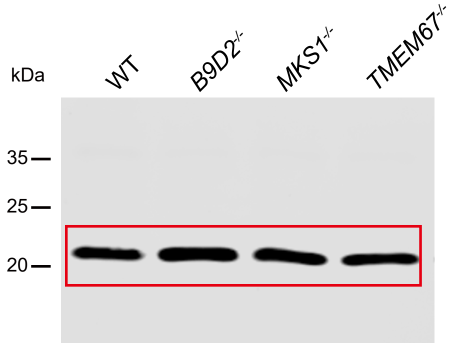

Antibody: FLAG

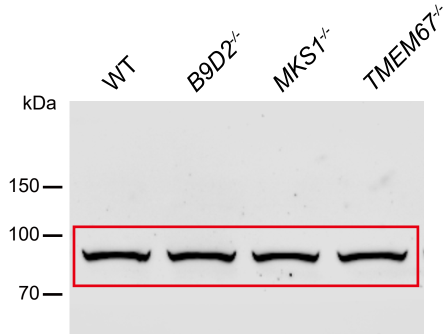

Antibody: FLAG

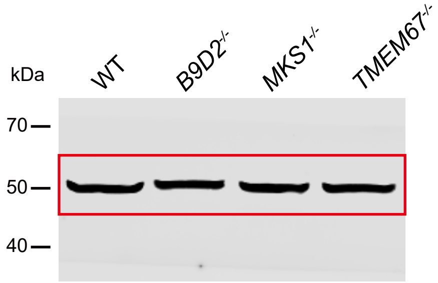

Antibody: α-Tub

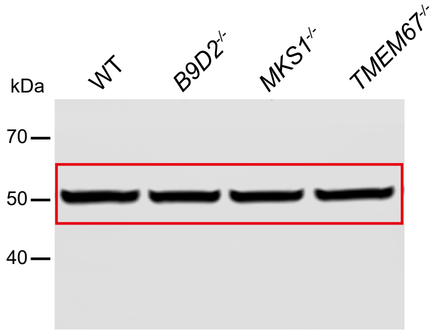

Antibody: α-Tub

Full unedited blot for Supplemental Figure 6 B

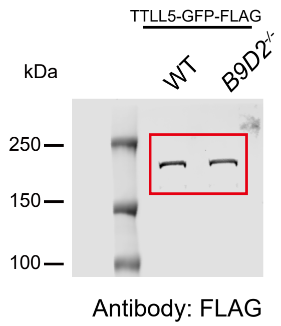

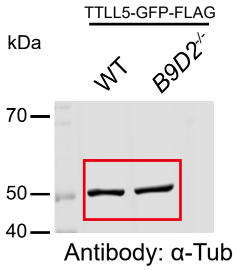

Full unedited blot for Supplemental Figure 6 F

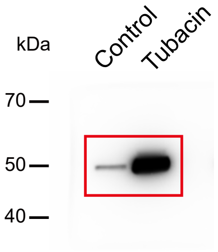

Antibody: Ace-Tub

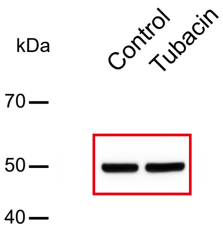

Antibody: α-Tub

Full unedited blot for Supplemental Figure 6 J

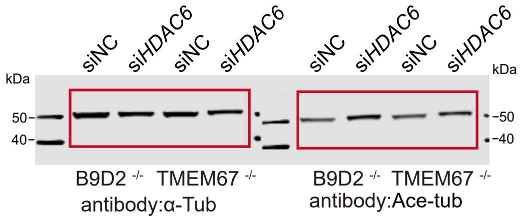

Full unedited blot for Supplemental Figure 8 A

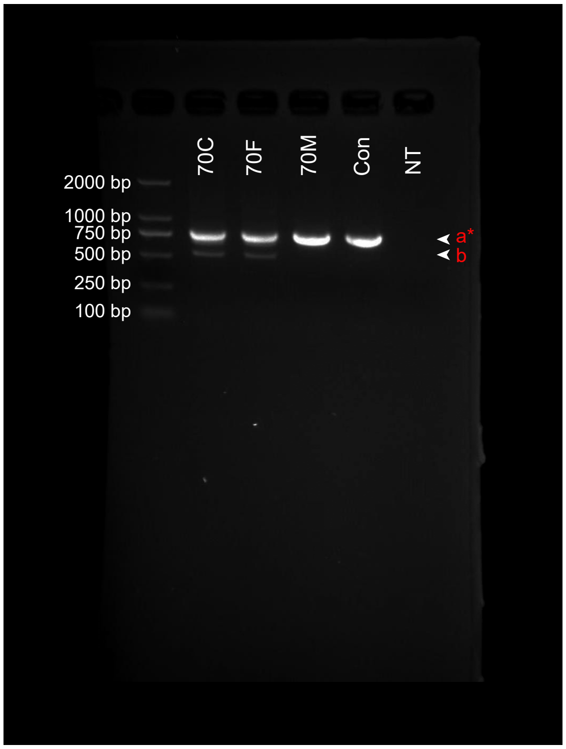

Supplement: Unedited blot and gel images [file jci-136-196365-s105.pdf]
